# Virtual Reality-Based Cognitive and Physical Interventions in Cognitive Impairment: A Network Meta-Analysis of Immersion Level Effects

**DOI:** 10.3390/bs15121610

**Published:** 2025-11-22

**Authors:** Wanyi Li, Wei Gao, Xiangyang Lin

**Affiliations:** College of Physical Education and Sport Science, Fujian Normal University, Minhou County, Fuzhou 350117, China

**Keywords:** virtual reality, cognitive training, physical activity, immersion level, mild cognitive impairment, dementia, executive function, network meta-analysis

## Abstract

Virtual reality (VR) has emerged as an innovative platform for delivering cognitive and physical training to individuals with cognitive impairment. However, the differential effectiveness of fully immersive versus partially immersive VR interventions remains unclear. This network meta-analysis aimed to evaluate how immersion level influences cognitive, motor, and functional outcomes in neurodegenerative populations. A systematic search of PubMed, Embase, Cochrane Library, and Web of Science up to October 2025 identified 20 randomized controlled trials involving 1382 participants with mild cognitive impairment (MCI) or dementia. Interventions were categorized into four groups: (1) fully immersive VR (head-mounted displays), (2) partially immersive VR (screen-based or motion-capture systems), (3) active control (traditional cognitive or physical training), and (4) passive control (usual care or health education). Outcomes included the Mini-Mental State Examination (MMSE), Montreal Cognitive Assessment (MoCA), Trail Making Test (TMT), Digit Span Test (DST), Timed Up and Go (TUG), and Instrumental Activities of Daily Living (IADL). Standardized mean differences (SMDs) and surface under the cumulative ranking curve (SUCRA) values were calculated using RevMan 5.4 and Stata 18.0. Fully immersive VR significantly improved global cognition compared to passive control (MMSE: SMD = 0.51, 95% CI [0.06, 0.96]), while partially immersive VR showed superior effects on executive function versus active control (TMT-B: SMD = −1.29, 95% CI [−2.62, −0.93]) and on motor function (TUG: SMD = −0.59, 95% CI [−1.11, −0.08]). In MoCA performance, both VR modalities outperformed traditional interventions (SUCRA: fully immersive = 76.0%; partially immersive = 84.8%). SUCRA rankings suggest that fully immersive VR is optimal for memory and foundational cognition (81.7%), whereas partially immersive VR performs best for executive function (98.9%). These findings indicate that the efficacy of VR-based cognitive or physical–cognitive interventions is modulated by immersion level. Tailoring VR modality to specific cognitive domains may optimize rehabilitation outcomes in MCI and dementia care.

## 1. Introduction

Dementia is a multifactorial syndrome characterized by progressive cognitive decline that impairs daily functioning and social participation (Gale et al., 2018). According to the World Health Organization, dementia is the seventh leading cause of death globally, affecting over 55 million people, with projections reaching 139 million by 2050 (World Health Organization, 2021). Alzheimer’s disease (AD) is the most common form of dementia, accounting for approximately 60–80% of cases (Anonymous, 2024), and mild cognitive impairment (MCI) represents a high-risk prodromal stage, where cognitive deficits emerge while daily functions remain largely intact (Gauthier et al., 2006). Individuals with MCI are nearly ten times more likely to progress to dementia annually, highlighting the need for early and effective interventions (Belleville et al., 2017).

Although pharmacological therapies exist, their effects are modest and often accompanied by adverse reactions. Consequently, non-pharmacological strategies such as physical exercise, cognitive training, and music therapy have gained prominence (Fu & Compton, 2021). Physical activity in particular provides dual benefits—enhancing both cognitive function and emotional well-being (Nuzum et al., 2020). Yet traditional interventions often suffer from low engagement, limited ecological validity, and poor adherence (Olazarán et al., 2010).

Virtual reality (VR) offers a potential solution by creating simulated, three-dimensional environments that combine cognitive and physical engagement. In cognitive rehabilitation, VR delivers multisensory, interactive experiences that enhance motivation and stimulate neuroplasticity in older adults with cognitive impairment (Riva & Serino, 2020; Tieri et al., 2018). Evidence indicates that VR-based interventions improve cognition, motor ability, emotional well-being, and activities of daily living, making them highly suitable for MCI and AD populations (Papaioannou et al., 2022; Q. Yang et al., 2025). A critical determinant of VR efficacy is the level of immersion, defined by the intensity of sensory feedback and user interaction. Fully immersive VR systems (e.g., head-mounted displays, HMDs) engage whole-body movements and spatial navigation tasks that promote physical activation, while partially immersive systems (e.g., screen- or tablet-based platforms) primarily involve seated cognitive tasks with limited kinesthetic input. Although both meet the WHO’s definition of bodily movement (World Health Organization, 2020), they differ substantially in motor intensity, attentional load, and cognitive engagement. Additionally, VR-based neuropsychological assessments—such as virtual supermarkets, route navigation, and cooking simulations—offer ecologically valid environments that enhance diagnostic sensitivity and cognitive testing accuracy (Ijaz et al., 2019; Miskowiak et al., 2022; K. Zhu et al., 2022). Thus, the diversity in VR immersion levels may partly explain the inconsistent findings reported in current studies (Li et al., 2011).

Nevertheless, Substantial heterogeneity persists across systems, tasks, and target populations (Gómez-Cáceres et al., 2023; Ren et al., 2024). For instance, while some studies demonstrate the benefits of immersive VR for spatial memory and hippocampal activation in MCI patients (Serino et al., 2017), others report no significant cognitive advantage over traditional methods (Delbroek et al., 2017). Furthermore, research has expanded to investigate broader neural and cognitive effects, such as enhanced fronto-occipital functional connectivity and visuospatial performance (Kang et al., 2021), reductions in depressive symptoms and improvements in global cognition (S. Zhu et al., 2021), and gains in executive function and mobility accompanied by enhanced attention-related cortical activity (Thapa et al., 2020). Collectively, these findings underscore that although VR interventions hold strong therapeutic potential, variability in immersion level and task design continues to challenge direct comparisons and interpretation of results. Preliminary evidence indicates that fully immersive VR may offer advantages in activating attentional systems, facilitating multisensory integration, and enhancing contextual learning (S. Zhu et al., 2021). However, whether it is categorically more effective remains uncertain, as some studies report potential drawbacks such as cybersickness, cognitive fatigue, or sensory overload among older adults with cognitive impairment (Breves & Stein, 2023). Furthermore, the majority of published VR intervention trials in cognitive impairment have been conducted in East Asian populations, with limited representation from Western or multi-ethnic cohorts. This geographic concentration may affect the generalizability of findings due to cultural, technological, and healthcare system differences. Therefore, the present study also highlights the need for cross-cultural validation and replication of VR-based cognitive rehabilitation research in more diverse samples.

Most previous meta-analyses have used traditional pairwise comparisons, which evaluate only two interventions at a time based solely on direct head-to-head evidence. In contrast, network meta-analysis (NMA) integrates both direct evidence (from studies directly comparing two interventions) and indirect evidence (from comparisons across a shared control group), thereby allowing simultaneous estimation of the relative effectiveness among multiple VR modalities. Given the wide variability in immersion levels across VR interventions, an NMA approach is particularly suited to determine the comparative efficacy of fully versus partially immersive VR. Accordingly, the present study systematically compared four intervention types—fully immersive VR, partially immersive VR, active control (traditional cognitive or physical training), and passive control (usual care or health education)—in individuals with mild cognitive impairment and dementia. We hypothesized that the level of immersion would modulate the therapeutic efficacy of VR-based interventions, with fully immersive VR producing greater improvements in global and memory-related cognition, and partially immersive VR showing superior effects in executive and motor functions. Surface under the cumulative ranking curve (SUCRA) values were used to evaluate the relative efficacy across cognitive, motor, and psychosocial outcomes.

Based on these findings, we propose a clinically applicable “precision immersion” framework for VR-based cognitive–physical rehabilitation, shifting the research focus from “whether VR is effective” to “how VR can be optimally effective.” This framework is grounded in established neurocognitive rehabilitation models emphasizing individualized and adaptive intervention strategies. For example, Guzmán et al. developed an adaptive VR rehabilitation system with personalized task difficulty (Guzmán et al., 2024); Faria et al. implemented customized web-based cognitive training across domains (Faria et al., 2018); and Seelye et al. highlighted the “person–task–environment” fit model for optimizing outcomes (Seelye et al., 2012). Together, these studies support the precision immersion framework as an extension of precision rehabilitation theory—linking immersion level, task complexity, and individual cognitive phenotype to maximize neural engagement and therapeutic efficacy.

## 2. Methods

### 2.1. Literature Search Strategy

A systematic search was conducted in PubMed, Embase, Cochrane Library, and Web of Science from database inception to October 2025. The search strategy combined Medical Subject Headings (MeSH) and free-text terms, including “virtual reality” OR “VR”, “immersive technology”, “mild cognitive impairment” OR “MCI”, “dementia”, “Alzheimer’s disease”, “cognitive function”, “motor function”, “activities of daily living”, and “emotion”. The strategy was independently developed by two reviewers (W.G. and W.L.) and verified by a third researcher (X.L.). Additionally, reference lists of relevant reviews and primary studies were manually screened to identify potentially eligible records. This study followed the Preferred Reporting Items for Systematic Reviews and Meta-Analyses (PRISMA) guidelines for study identification, inclusion, exclusion, and data extraction (Page et al., 2021). Two reviewers independently extracted data using a standardized form covering study information (author, year, disease type, population source); participant characteristics (sample size, age, sex, education); intervention details (cycle, frequency, VR type, immersion level, and experimental protocol); control conditions (type and protocol); follow-up duration; and primary outcome measures. Discrepancies were resolved by discussion or consultation with a third reviewer. The complete PRISMA checklist and extracted dataset are available in the Supplementary Materials.

Although the search strategy placed no initial date restrictions to maximize comprehensiveness, the included studies were all published from 2017 onward. This reflects the field’s maturation, as the widespread clinical adoption of standardized, consumer-grade virtual reality hardware (e.g., HMDs) began around this time, ensuring technological comparability across the analyzed interventions.

### 2.2. Selection and Exclusion Criteria

#### 2.2.1. Inclusion Criteria

Inclusion criteria were based on the Population, Intervention, Comparison, Outcome, Study design (PICOS) framework:

(a) Population: Individuals with cognitive impairment, including MCI or dementia, diagnosed using standardized diagnostic criteria.
(b) Intervention: VR interventions, including fully immersive VR (e.g., HMDs with motion sensors) and partially immersive VR (e.g., screen-based or desktop-interactive platforms). Interventions may involve cognitive, physical, or combined physical–cognitive tasks.
(c) Comparison: Active control (e.g., traditional cognitive or physical training) or passive control (e.g., usual care or no intervention).
(d) Outcomes: Studies were required to report at least one of the following quantitative outcomes:
  Cognitive outcomes: Mini-Mental State Examination (MMSE), Montreal Cognitive Assessment (MoCA), Cognitive Abilities Screening Instrument (CASI), or other scales assessing global cognition, memory, language, and attention;
  Executive function outcomes: Trail Making Test (TMT)-A/B, Digit Span Test (DST)-forward/backward, Symbol Digit Substitution Test (SDST), Stroop Color-Word Test (SCWT), Verbal Fluency Test (VFT), Frontal Assessment Battery (FAB), and others evaluating cognitive flexibility, working memory, task switching, and inhibition;
  Motor function outcomes: Timed Up and Go (TUG), grip strength, Fried frailty phenotype (FFP), etc.;
  Daily functioning and psychosocial outcomes: Instrumental Activities of Daily Living (IADL), SGDS (Geriatric Depression Scale), other validated tools assessing mood, depressive symptoms, or emotional well-being.
(e) Study design: Only randomized controlled trials (RCTs) were included.

#### 2.2.2. Exclusion Criteria

Studies were excluded if they met any of the following criteria:

(a) Non-RCT designs (e.g., observational studies, pre-post single group designs, case series);
(b) Interventions lacking a clearly defined VR component or using simple video playback;
(c) Participants were healthy individuals, had severe dementia, or other neurological/psychiatric disorders;
(d) Incomplete outcome data, or the effect size could not be calculated;
(e) Non-English full texts, or abstract-only/commentary publications.

#### 2.2.3. Classification of VR Interventions

VR interventions were categorized according to the level of immersion and the type of display device, which are considered fundamental dimensions in defining VR systems (Makransky et al., 2019; N. Yu et al., 2025).

(a) Fully immersive VR refers to systems that completely immerse the user in a virtual environment through HMDs (e.g., HTC Vive, Oculus Rift) or motion-based immersive systems (e.g., Computer Assisted Rehabilitation Environment (CAREN). These systems provide 360° stereoscopic vision, motion tracking, and auditory feedback, effectively isolating users from the physical world and generating a strong sense of presence and embodiment.
(b) Partially immersive VR, in contrast, presents the virtual environment on conventional flat displays such as monitors, TVs, projectors, or tablets. Users interact via standard input devices (e.g., mouse, keyboard, or limited motion sensors), maintaining partial awareness of their real surroundings. This configuration provides limited sensory feedback and weaker spatial immersion compared with fully immersive systems.

Notably, both VR types may incorporate physical tasks such as balance training, walking, or upper limb movement, enabling a form of virtual physical activity relevant to cognitive.

The inclusion criteria required studies to provide sufficient methodological detail to classify the VR intervention by immersion and interactivity; trials using non-immersive programs, hybrid systems, or those lacking clear descriptions of device/interaction features were excluded to ensure conceptual and methodological consistency in comparing interventions that differ primarily in immersion level.

### 2.3. Risk of Bias and Quality Assessment

The risk of bias in the included RCTs was assessed using the Cochrane Risk of Bias 2.0 tool, covering five domains: randomization process, deviations from intended interventions, missing outcome data, outcome measurement, and selective reporting. Each domain was rated as “low-risk”, “some concerns”, or “high-risk”. Assessment was conducted independently by two reviewers, with disagreements resolved by a third party.

### 2.4. Data Synthesis and Statistical Analysis

Both traditional and network meta-analyses were performed: Traditional meta-analysis: A random-effects model was used to calculate standardized mean differences (SMDs) and 95% confidence intervals (CIs). Heterogeneity was assessed using the I^2^ statistic, with I^2^ > 50% considered moderate to high heterogeneity. Network meta-analysis: Conducted under a frequentist framework to compare the relative effects of fully immersive VR, partially immersive VR, active control, and passive control. The frequentist approach was selected because it enables direct estimation of treatment effects and ranking probabilities without requiring prior distributions, which is particularly suitable for datasets with a moderate number of studies and limited prior information. This framework also ensures comparability with previous neurorehabilitation NMAs and provides computational simplicity and reproducibility. While Bayesian models offer greater flexibility in incorporating prior assumptions, the frequentist model was preferred for its transparency and consistency with established NMA practices in similar research contexts. SUCRA values were used to rank the effectiveness of each intervention, with higher values indicating greater efficacy. Consistency test: Node-splitting methods were applied to evaluate consistency between direct and indirect comparisons. Subgroup and sensitivity analyses: Subgroup analysis was performed based on the severity of cognitive impairment; sensitivity analysis excluded high-risk studies to test the robustness of findings. Publication bias: Given that the number of studies for each comparison was below the recommended threshold (*n* < 10) for reliable analysis, formal assessment using funnel plots and Egger’s test was not performed, as per methodological guidelines. All analyses were conducted using Stata 18.0 and RevMan 5.3.

This review adhered strictly to the PRISMA extension statement for network meta-analyses and was prospectively registered with PROSPERO (CRD420251040288).

## 3. Results

### 3.1. Study Selection and Quality Assessment

**Figure 1:**
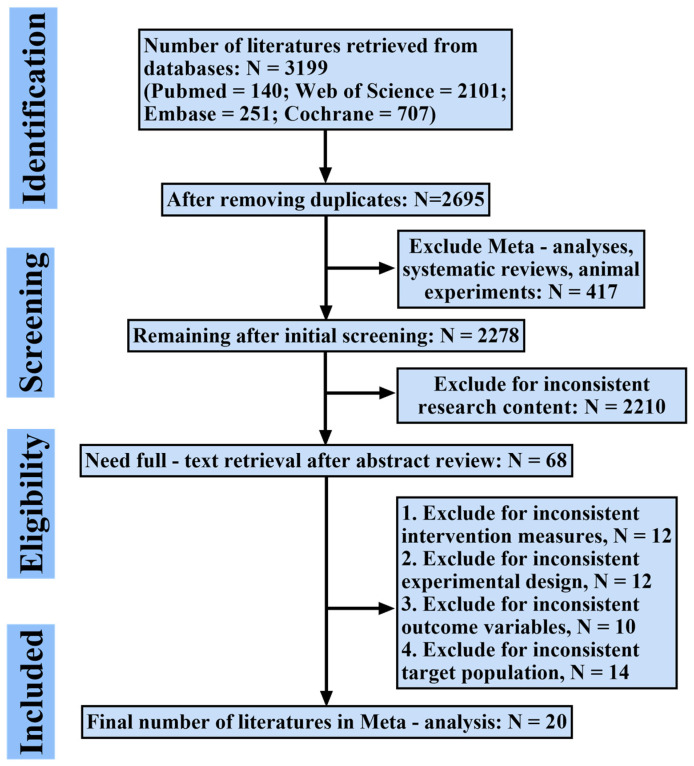
Flowchart of study selection process based on PRISMA guidelines. This figure illustrates the number of records identified, screened, excluded, and included at each stage.

**Figure 2:**
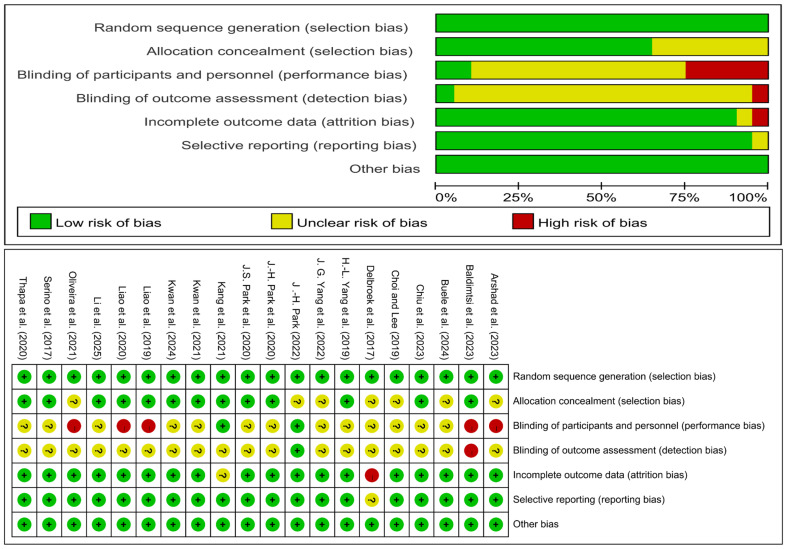
Assessment result of the risk of bias. There are three ratings, green for low risk of bias, yellow for uncertain risk of bias, and red for high risk of bias. The included studies are: Arshad et al. (2023), Baldimtsi et al. (2023), Buele et al. (2024), Chiu et al. (2023), Choi and Lee (2019), Delbroek et al. (2017), H.-L. Yang et al. (2019), J. G. Yang et al. (2022), J.-H. Park (2022), J.-H. Park et al. (2020), J. S. Park et al. (2020), Kang et al. (2021), Kwan et al. (2021), Kwan et al. (2024), Liao et al. (2019), Liao et al. (2020), Li et al. (2025), Oliveira et al. (2021), Serino et al. (2017), Thapa et al. (2020).

The systematic literature search identified 3199 records. After duplicate removal and a rigorous screening process, 20 RCTs were ultimately included in the network meta-analysis. The study selection process, detailed in the PRISMA flowchart (Figure 1) and Supplementary Table S1, was characterized by the exclusion of records primarily due to non-RCT designs, ineligible interventions, or incompatible populations. All included studies were published in English and demonstrated acceptable methodological quality, with the results of the risk of bias assessment presented in Figure 2.

### 3.2. Characteristics of Included Studies

A total of 20 RCTs (*n* = 1382 participants) published between 2017 and 2025 were included. Key characteristics are summarized in Table 1. Participants were older adults (mean ages 62.9–88.9 years), predominantly female (median = 70.1%). Most studies (15/20) targeted individuals with MCI, while others included AD, cognitive frailty, or prodromal dementia.

Geographically, studies were concentrated in East Asia—accounting for 75% of all trials, with 8 from mainland China/Hong Kong/Taiwan and 7 from South Korea. This demographic concentration highlights the limited global representativeness of the current VR evidence base. Recruitment was primarily from community centers, day-care institutions, or clinics. Education levels were generally low (4.8–9.9 years), although nearly half of studies did not report this variable.

VR intervention protocols varied widely, yet a common pattern emerged: the median regimen involved 60-min sessions, conducted twice weekly over an 8-week period. However, sample sizes varied widely, potentially introducing heterogeneity. A comparative overview of the VR systems revealed a nearly equal split between fully immersive (12 trials) and partially immersive (8 trials) approaches (see Table S2 for details), with Oculus and HTC Vive being the predominant HMDs. Task designs were diverse, encompassing functional daily living simulations, multi-domain cognitive training, and dual-task paradigms, with several studies (*n* = 7) incorporating adaptive difficulty.

Regarding control groups and outcomes, a balanced design was observed, with half of the trials using active comparators and the other half using passive controls. The most frequently assessed domains were global cognition (MMSE/MoCA) and executive function (TMT/DST). A critical limitation across the literature was the scarcity of long-term data, with only two studies reporting follow-up assessments beyond the immediate intervention period. The detailed results of the meta-regression are presented in Table S3.

### 3.3. Cognitive Function Outcomes

#### 3.3.1. MMSE

Nine studies assessed MMSE scores across four intervention types: fully immersive VR (Group A), partially immersive VR (Group B), active controls (Group C), and passive controls (Group D).

**Figure 3:**
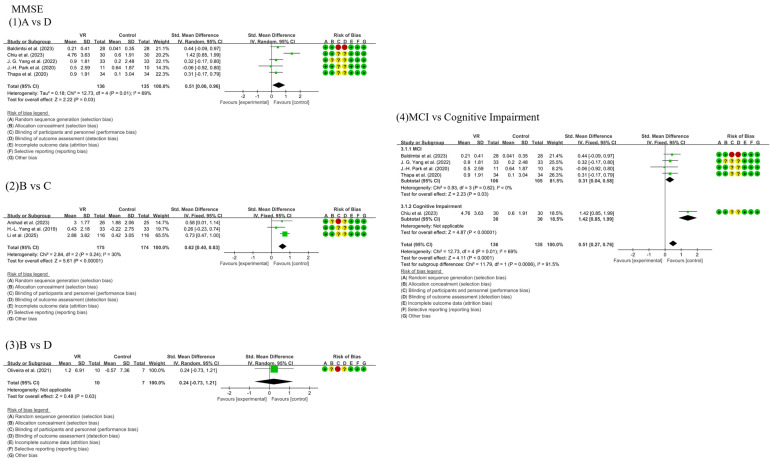
Forest plots of MMSE outcomes across intervention comparisons and subgroup analysis. (**1**) Comparison between Fully Immersive VR (A) and Passive Control (D); (**2**) Comparison between Partially Immersive VR (B) and Active Control (C); (**3**) Comparison between Partially Immersive VR (B) and Passive Control (D); (**4**) Subgroup analysis based on the severity of cognitive impairment; Effect sizes are presented as SMDs with 95% CIs. A positive SMD indicates greater improvement in the VR or active intervention group compared to the control. The included studies are: Baldimtsi et al. (2023), Chiu et al. (2023), J. G. Yang et al. (2022), J.-H. Park et al. (2020), Thapa et al. (2020), Arshad et al. (2023), H.-L. Yang et al. (2019), Li et al. (2025), Oliveira et al. (2021).

As shown in Figure 3, in conventional meta-analysis, the comparison between Group A and Group D (5 studies, *n* = 271) showed that fully immersive VR significantly improved MMSE scores compared to passive control (SMD = 0.51, 95% CI [0.06, 0.96], *p* = 0.03), with observed heterogeneity (I^2^ = 69%, *p* = 0.01). Sensitivity analysis excluding one outlier study (with severe baseline impairment) eliminated heterogeneity (I^2^ = 0%) while the effect size remained stable, confirming greater benefit for MCI participants. The comparison between Group B and Group C (3 studies, *n* = 349) showed partially immersive VR significantly outperformed active controls (SMD = 0.62, 95% CI [0.40, 0.83], *p* < 0.00001) with low heterogeneity (I^2^ = 30%). The single-study comparison between Group B and Group D did not reach significance (SMD = 0.24, 95% CI [−0.73, 1.21], *p* = 0.63).

**Figure 4:**
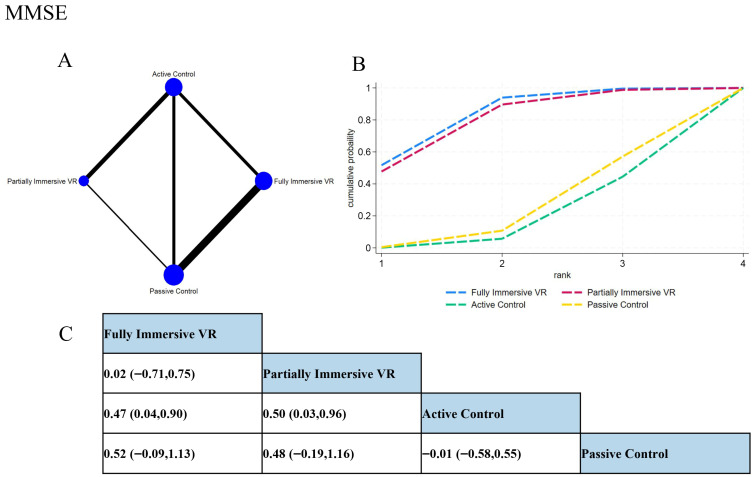
Network meta-analysis results of MMSE outcomes. (**A**) Network plot showing direct and indirect comparisons; node size reflects sample size, and edge thickness represents the number of comparisons. (**B**) SUCRA ranking plot of each intervention, where Surface Under the Cumulative Ranking Curve indicates better relative efficacy. (**C**) League table presenting the pairwise SMDs and 95% CIs for all comparisons among interventions.

Network meta-analysis (Figure 4) indicated fully immersive VR demonstrated superior performance, showing significant advantage over passive controls (SMD = 0.50, 95% CI [0.09, 0.91]) and positive albeit non-significant effect versus active controls (SMD = 0.51, 95% CI [−0.08, 1.10]). Partially immersive VR also significantly outperformed active controls (SMD = 0.50, 95% CI [0.03, 0.96]). The difference between the two VR modalities was negligible (SMD = 0.01, 95% CI [−0.69, 0.72]).

SUCRA rankings supported these findings: fully immersive VR ranked highest (81.7%), followed by partially immersive VR (78.7%), both substantially outperforming active (16.8%) and passive controls (22.8%). These results suggest that VR interventions offer significant cognitive benefits for patients with MCI and AD, with clinical selection tailorable based on individual patient characteristics and equipment availability given their comparable efficacy on global cognition.

#### 3.3.2. MoCA

A total of 10 studies assessed changes in MoCA scores. In conventional meta-analysis (Figure S1), partially immersive VR (Group B) demonstrated a significant cognitive benefit over active controls (Group C) (5 studies, *n* = 444; SMD = 0.40, 95% CI [0.21, 0.59], *p* < 0.0001; I^2^ = 0%). In contrast, fully immersive VR (Group A) showed a positive but non-significant trend versus active controls (3 studies, *n* = 77; SMD = 0.30, 95% CI [−0.15, 0.75], *p* = 0.20). A single study (*n* = 293) found fully immersive VR superior to passive control (Group D) (SMD = 0.24, 95% CI [0.01, 0.47], *p* = 0.04), while another single study found no significant benefit of partially immersive VR over passive control (SMD = 0.24, 95% CI [−0.64, 1.12], *p* = 0.60).

The network meta-analysis (Figure S2) reinforced the advantage of partially immersive VR, which significantly outperformed active controls (SMD = 0.40, 95% CI [0.21, 0.58]). Fully immersive VR showed a marginally significant benefit over passive controls (SMD = 0.23, 95% CI [0.01, 0.45]). Critically, no significant difference was found between the two VR modalities (SMD = 0.08, 95% CI [−0.35, 0.51]).

SUCRA rankings positioned partially immersive VR as the top intervention (84.8%; 63.9% probability of being best), followed by fully immersive VR (76.0%; 35.4% probability of being best). Both VR modalities substantially outperformed active (SUCRA = 13.7%) and passive controls (SUCRA = 25.5%), underscoring the efficacy of VR-based strategies for enhancing cognitive function on the MoCA.

#### 3.3.3. CASI

The network structure for CASI did not form a closed loop, precluding network meta-analysis, as only two independent direct comparisons (B vs. C; A vs. D) were available. Conventional meta-analysis (Figure S3) indicated that partially immersive VR (Group B) was significantly more effective than active controls (Group C) (SMD = 0.54, 95% CI [0.28, 0.80], *p* < 0.0001). Fully immersive VR (Group A) showed a very large effect size compared to passive controls (Group D) (SMD = 1.65, 95% CI [1.06, 2.24], *p* < 0.00001), suggesting potential particular benefit for more severely impaired patients. As each comparison was informed by a single study, heterogeneity could not be computed. Future studies are needed to expand these intervention nodes for network-level integration.

### 3.4. Executive Function Outcomes

#### 3.4.1. TMT-A

Five studies assessed TMT-A, which measures visual scanning and attentional switching. In conventional meta-analysis (Figure S4), partially immersive VR (Group B) showed a significant advantage over active controls (Group C) (*n* = 86; SMD = −1.40, 95% CI [−1.88, −0.93], *p* < 0.00001; I^2^ = 0%). Fully immersive VR (Group A) showed a non-significant trend toward improvement over passive controls (Group D) (*n* = 134; SMD = −0.32, 95% CI [−0.66, 0.03], *p* = 0.07).

Network meta-analysis (Figure S5) revealed a clear advantage for partially immersive VR, which significantly outperformed active controls (SMD = −1.40, 95% CI [−1.88, −0.93]), fully immersive VR (SMD = −1.26, 95% CI [−1.87, −0.65]), and passive controls (SMD = −1.57, 95% CI [−2.21, −0.94]). Fully immersive VR did not show significant differences versus either control group (SMD ≥ −0.31, *p* > 0.05).

SUCRA rankings positioned partially immersive VR as the top intervention (100%), followed by fully immersive VR (57.9%), active control (34.2%), and passive control (7.9%). These findings suggest partially immersive VR is the optimal modality for improving processing speed, likely due to its precise targeting of visual search capabilities.

#### 3.4.2. TMT-B

TMT-B assesses cognitive flexibility under complex executive load. Conventional meta-analysis (Figure S6) demonstrated that fully immersive VR (Group A) significantly reduced completion time versus passive controls (Group D) (2 studies, *n* = 124; SMD = −0.48, 95% CI [−0.84, −0.12], *p* = 0.009). Partially immersive VR (Group B) showed a large, albeit non-significant, effect versus active controls (Group C) (2 studies, *n* = 86; SMD = −1.29, 95% CI [−2.62, 0.03], *p* = 0.06). A single study comparing Group A with Group C (*n* = 34) reported no significant difference (SMD = −0.47, *p* = 0.18).

In network meta-analysis (Figure S7), partially immersive VR significantly outperformed both active (SMD = −1.29, 95% CI [−2.14, −0.44]) and passive controls (SMD = −1.74, 95% CI [−3.03, −0.45]), and showed a potential advantage over fully immersive VR (SMD = −1.18, 95% CI [−2.37, 0.01], *p* = 0.052). SUCRA rankings identified partially immersive VR as the top intervention (98.9%). Although fully immersive VR outperformed passive controls (SMD = −0.56, 95% CI [−1.33, 0.20]), its clinical relevance was moderate. The network synthesis provides robust support for partially immersive VR as the optimal strategy for enhancing cognitive flexibility.

#### 3.4.3. SDST

For information processing speed (SDST), two studies (*n* = 134) showed fully immersive VR (Group A) significantly improved performance versus passive controls (Group D) (SMD = 1.30, 95% CI [0.92, 1.68], *p* = 0.01), despite substantial heterogeneity (I^2^ = 86%) (Figure S8). Network meta-analysis was not feasible due to the limited number of comparison nodes.

#### 3.4.4. DST-Forward

Regarding short-term memory span (DST-forward), conventional meta-analysis showed a positive trend for partially immersive VR (Group B) over active controls (Group C) (n = 55; SMD = 0.87, 95% CI [−0.03, 1.78], *p* = 0.06; I^2^ = 60%), while fully immersive VR (Group A) did not significantly differ from passive controls (n = 370; SMD = 0.13, 95% CI [−0.07, 0.34], *p* = 0.20; I^2^ = 0%) (Figure S9).

Network meta-analysis (Figure S10) indicated both partially immersive (SMD = 0.92, 95% CI [0.35, 1.49]) and fully immersive VR (SMD = 0.87, 95% CI [0.20, 1.54]) significantly outperformed active controls, with no significant difference between them (SMD = 0.05, 95% CI [−0.83, 0.93]). SUCRA rankings confirmed VR superiority, with fully immersive (76.2%) and partially immersive VR (73.7%) together having a 94.0% combined probability of being the best intervention.

#### 3.4.5. DST-Backward

For working memory (DST-backward), partially immersive VR (Group B) showed a significant advantage over active controls (Group C) (2 studies, *n* = 101; SMD = 0.56, 95% CI [0.16, 0.96], *p* = 0.006; I^2^ = 0%) (Figure S11). In contrast, fully immersive VR (Group A) did not differ significantly from passive controls (*n* = 314; SMD = −0.04, 95% CI [−0.26, 0.18], *p* = 0.71). Network meta-analysis was not performed due to the unconnected network.

#### 3.4.6. SCWT

For cognitive inhibition (SCWT), neither fully immersive VR versus active controls (n = 34; SMD = 0.13, 95% CI [−0.55, 0.80], *p* = 0.71) nor versus passive controls (*n* = 293; SMD = 0.08, 95% CI [−0.15, 0.31], *p* = 0.51) showed significant effects (Figure S12). Network meta-analysis was not conducted.

#### 3.4.7. VFT

For verbal fluency (VFT), no significant benefits were found for partially immersive VR versus active controls (*n* = 71; SMD = 0.23, 95% CI [−0.95, 1.42], *p* = 0.70; I^2^ = 80%) or for fully immersive VR versus passive controls (*n* = 21; SMD = 0.00, 95% CI [−0.86, 0.86], *p* = 1.00) (Figure S13). Network analysis was not performed.

#### 3.4.8. FAB

For frontal lobe function (FAB), comparisons of partially immersive VR versus active controls (*n* = 20; SMD = 0.43, 95% CI [−0.46, 1.32], *p* = 0.34) and versus passive controls (*n* = 17; SMD = 0.19, 95% CI [−0.78, 1.16], *p* = 0.70) showed no significant effects (Figure S14). Network meta-analysis was not feasible.

### 3.5. Physical Function Outcomes

#### 3.5.1. TUG

For dynamic balance and gait safety (TUG), conventional meta-analysis (Figure S15) indicated partially immersive VR (Group B) significantly outperformed active controls (Group C) in one study (*n* = 60; SMD = −0.59, 95% CI [−1.11, −0.08], *p* = 0.02). In contrast, fully immersive VR (Group A) showed no significant improvement over passive controls (Group D) (*n* = 293; SMD = −0.18, 95% CI [−0.41, 0.05], *p* = 0.13).

Network meta-analysis (Figure S16) showed a consistent, though non-significant, trend for partially immersive VR over active controls (SMD = −0.44, 95% CI [−0.93, 0.04]). SUCRA rankings identified partially immersive VR as the most effective intervention (86.1%), followed by fully immersive VR (62.4%), passive control (27.3%), and active control (24.3%), suggesting its priority for improving TUG performance.

#### 3.5.2. Grip Strength

Regarding grip strength, conventional meta-analysis (Figure S17) showed partially immersive VR (Group B) significantly improved outcomes compared to active controls (Group C) (*n* = 60; SMD = 0.53, 95% CI [0.02, 1.05], *p* = 0.04). Fully immersive VR (Group A) showed no significant difference versus passive controls (Group D) (*n* = 68; SMD = 0.29, 95% CI [−0.19, 0.77], *p* = 0.23). Network meta-analysis was not conducted due to an unconnected network, but the direct evidence supports partially immersive VR for enhancing grip strength.

#### 3.5.3. FFP

For physical frailty (FFP), conventional meta-analysis (Figure S18) showed fully immersive VR (Group A) significantly reduced frailty versus passive controls (Group D) (*n* = 293; SMD = −0.31, 95% CI [−0.54, −0.08], *p* = 0.008), but showed no effect versus active controls (Group C) (*n* = 17; SMD = 0.00, 95% CI [−0.95, 0.95], *p* = 1.00). Network meta-analysis was not feasible due to missing data on partially immersive VR and limited network connectivity. Future research should include these comparisons.

### 3.6. Emotional and Quality of Life Outcomes

#### 3.6.1. IADL

For independent living capacity (IADL), conventional meta-analysis (Figure S19) revealed non-significant trends favoring fully immersive VR over active controls (*n* = 60; SMD = 0.38, 95% CI [−0.13, 0.90], *p* = 0.14; I^2^ = 0%) and partially immersive VR over passive controls (*n* = 49; SMD = 0.50, 95% CI [−0.08, 1.08], *p* = 0.09; I^2^ = 46%). Network meta-analysis was not performed due to an unconnected network.

#### 3.6.2. SGDS

For depressive symptoms (SGDS), conventional meta-analysis (Figure S20) showed no significant effects for fully immersive VR versus passive controls (*n* = 62; SMD = −0.16, 95% CI [−0.66, 0.34], *p* = 0.53) or versus active controls (*n* = 26; SMD = 0.53, 95% CI [−0.26, 1.32], *p* = 0.19). Network meta-analysis was not conducted due to a severely fragmented network lacking data for partially immersive VR.

## 4. Discussion

This network meta-analysis systematically compared the effects of four intervention modalities—fully immersive VR, partially immersive VR, active controls, and passive controls—on multiple cognitive and functional outcomes among individuals with cognitive impairment. The findings revealed that VR-based interventions, particularly those with varying levels of immersion, were consistently more effective than traditional interventions in enhancing global cognition, executive function, attention, mood, and daily functional capacity. Notably, partially immersive VR ranked first across several key outcomes (e.g., MoCA, TMT-A, TMT-B, TUG), suggesting its superior overall effectiveness.

### 4.1. Differential Effects of Immersion: Neural Mechanisms, Cognitive Domain Specificity, and Mental Health Links

This study provides the first comprehensive network-based evidence that the cognitive benefits of VR interventions are significantly influenced by the level of immersion—a finding that sheds light on the neural mechanisms underlying digital therapeutics. Fully immersive VR (via HMDs) demonstrated clear advantages in improving basic cognitive functions, such as those measured by the MMSE (SUCRA = 81.7%), suggesting a positive effect on orientation, recall, and attentional processes. These effects may stem from multisensory stimulation and ecologically valid interactions that enhance neural network integration and promote neuroplasticity.

Mechanistically, immersive VR may activate hippocampal–entorhinal pathways via spatial encoding, increase functional connectivity in the fronto-occipital network, and enhance low-frequency oscillations (e.g., Delta/Theta bands), facilitating improved spatial cognition and memory formation (Beanato et al., 2024; Howett et al., 2019; Kang et al., 2021; Z. Tang et al., 2022). This aligns with the observed improvements in MMSE scores, indicating that immersive environments strengthen memory encoding by simulating real-world spatial contexts and activating grid cells in the entorhinal cortex. Preserved foundational cognition may mitigate depression risk, given that dementia and MCI are linked to a substantially higher prevalence of depressive symptoms relative to cognitively normal older adults (Snowden et al., 2015).

Conversely, the MoCA results showed superior efficacy for partially immersive VR (SUCRA = 84.8%), which assesses higher-order cognitive domains such as executive function, naming, visuoconstruction, attention, and abstraction. As MoCA is more sensitive than MMSE (Ciesielska et al., 2016), the improvements are likely driven by enhanced visual attention and task-switching capacities. Partially immersive VR—delivered via large screens or motion-capture systems (whole-body exergames)—emphasizes motor-driven, rule-based learning and cognitive strategy engagement, which may better activate executive systems while minimizing cybersickness barriers to physical activity adherence.

We also observed outstanding effects of partially immersive VR in executive function domains, such as TMT-B and TUG (TMT-B SUCRA = 98.9%, SMD = −1.29), consistent with findings from D. Yu et al. (2023). These effects may be attributed to the system’s emphasis on motor–cognitive integration, goal-directed actions (e.g., virtual obstacle courses), and cognitive flexibility, likely involving the dorsolateral prefrontal cortex (DLPFC) and anterior cingulate cortex (ACC) (H. Tang et al., 2016). While fully immersive VR offers stronger sensory realism, it may also cause simulator sickness or cognitive overload in some MCI patients (Saredakis et al., 2020), undermining Physical Activity (PA) participation and mental well-being—especially in older or neurologically vulnerable groups.

Previous studies have highlighted that user acceptability and cognitive load are critical determinants of PA sustainability in VR interventions (Wu et al., 2023). Partially immersive VR may strike a better balance between sensory engagement and cognitive comfort, promoting long-term PA adherence. These findings emphasize that tailoring PA modalities to individual profiles (e.g., sensory tolerance, motor capacity) is essential for optimizing mental health outcomes, particularly in neurodiverse or aging populations where “one-size-fits-all” approaches fail. Ultimately, the differential effects observed across immersion levels underscore the importance of task-specific VR design. When VR programs include physically active and cognitively demanding components, they may yield broader functional benefits than passive or sedentary forms of VR, especially in populations at risk for both cognitive and psychological decline.

### 4.2. Advantages over Traditional Therapies: Enhancing Physical Activity Adherence

The SUCRA rankings provide compelling evidence for the clinical prioritization of VR interventions. In the primary outcome of MoCA, the combined probability of VR being the best intervention reached 99.3% (partially immersive VR: 63.9%; fully immersive VR: 35.4%), significantly outperforming active controls (13.7%) and passive controls (25.5%). This highlights VR’s unique ability to overcome the “adherence dilemma” in non-pharmacological interventions—a critical barrier for mental health gains in cognitively impaired populations.

Compared to traditional exercise or cognitive therapy, VR enhances motivation and engagement (Hill et al., 2017). Through multisensory integration and mirror neuron activation, VR boosts empowerment and confidence, encouraging participation in otherwise repetitive or abstract tasks (Bauer & Andringa, 2020; Rizzolatti & Fogassi, 2014). Our analysis suggests a three-fold mechanism by which VR overcomes adherence barriers: Motivational enhancement: Gamified movement (e.g., juice-making, shooting games (Thapa et al., 2020)) increases enjoyment and perceived self-efficacy, which are key predictors of mental well-being and sustained behavior change. Ecologically valid motor tasks (e.g., bus transfers (Kwan et al., 2024)) enhance functional relevance and support daily life coping skills, potentially reducing feelings of helplessness or disengagement. Personalized challenge: Adaptive difficulty (e.g., virtual kitchen tasks (Buele et al., 2024)) maintains a challenge-skill balance, which prevents boredom and frustration in cognitively declined individuals and supports the flow experience, an intrinsic motivational state closely linked with both cognitive stimulation and emotional regulation.

Moreover, partially immersive VR interventions frequently integrate body-based interactions—such as stepping, reaching, or turning—resembling light-intensity physical activity. These movement components may offer additional benefits by stimulating dopaminergic and serotonergic systems, which are associated with enhanced mood, motivation, and cognitive alertness (Meeusen, 2005). In this sense, VR serves not only as a cognitive rehabilitation tool but also as a multimodal physical–cognitive platform, aligning with the biopsychosocial model of intervention by addressing cognitive, emotional, and behavioral dimensions simultaneously.

Taken together, these findings suggest that immersion-level-specific VR interventions may outperform conventional therapies not only due to their technical novelty, but also due to their superior capacity to promote adherence, emotional resilience, and embodied cognitive engagement—all of which are crucial for long-term mental and functional health maintenance in individuals with neurodegenerative conditions.

### 4.3. Reconciling Contradictory Findings: From Surface Discrepancies to Precision Prescriptions

This study also revealed several apparent “contradictions” that underscore the complexity of intervention design and patient heterogeneity. Rather than inconsistencies, these patterns reflect task sensitivity, baseline stimulation levels, and neural specificity.

#### 4.3.1. The “Effective vs. Passive Control, but Not vs. Active Control” Paradox

In MMSE outcomes, fully immersive VR showed significant benefits over passive controls (SMD = 0.51, *p* = 0.03), but not over active controls. This disparity may be explained by a threshold effect of baseline stimulation—passive control groups, typically exposed to minimal cognitive or physical engagement, exhibit greater observable improvement due to the novelty and intensity of VR training. In contrast, active control interventions (e.g., structured physical or cognitive therapy) may already elicit partial neurocognitive activation, narrowing the comparative effect size. Similar trends were noted by Delbroek et al., where even low-dose VR training (18–30 min/session) outperformed non-intervention but not structured comparators (Delbroek et al., 2017). From a mental health perspective, this pattern implies that VR may be particularly beneficial in under-stimulated populations or in settings where engagement and affective stimulation are lacking—highlighting its potential role in preventive digital enrichment for institutionalized or socially isolated individuals.

#### 4.3.2. No Difference Between VR Types: A Task-Specific Insight

In DST-forward outcomes, no significant difference was observed between the two VR modalities (SMD = 0.05, 95% CI [−0.83, 0.93]). This should not be interpreted as therapeutic equivalence, but rather as a reflection of task-specific ceiling effects. Working memory tasks like forward digit span primarily depend on prefrontal and parietal activation, requiring minimal immersive engagement. For such cognitively “narrow-band” tasks, the added sensory input of fully immersive VR may offer little marginal benefit over screen-based formats. These findings emphasize the importance of aligning VR modality selection with targeted cognitive domains and desired psychological states (e.g., arousal, engagement), supporting the development of task-matched protocols.

### 4.4. Clinical Translation: Stratified Implementation Framework

Building upon these findings, we propose a stratified implementation strategy based on cognitive phenotype, resource availability, and functional goals to guide the real-world application of VR. The core of this “precision immersion” approach is to first match the VR modality to the individual’s primary cognitive deficit, as identified through clinical assessment, and then tailor the specific tasks to their functional goals and setting.

In primary care and community settings, the focus is often on enhancing executive function and physical mobility for a diverse group. Partially immersive VR (e.g., Kinect or tablet-based systems) is recommended due to its accessibility and proven benefits in these domains. The implementation can be concretely guided by assessment: for patients with identified deficits in cognitive flexibility (e.g., poor TMT-B performance), a tablet-based “virtual supermarket” task can be prescribed to specifically target planning and task-switching. For those with mobility concerns, a group-based virtual exergame requiring participants to step over obstacles on a large screen can be deployed to simultaneously train balance, processing speed, and foster psychosocial engagement.

Memory clinics and specialty centers are poised to implement a more targeted, phenotype-driven approach. For patients diagnosed with memory-dominant MCI or early-stage AD—where the primary deficit lies in episodic memory and spatial orientation—fully immersive VR should be the intervention of choice. The clinical pathway here involves prescribing high-immersion scenarios, such as HMD-based route navigation training where patients practice finding their way in a virtual neighborhood, to directly stimulate hippocampal circuits. This can be extended beyond training to assessment, using standardized VR functional capacity assessments like completing a series of IADLs in a virtual kitchen, which provides a more sensitive measure of cognitive decline and rehabilitation progress specific to this patient phenotype.

Within nursing homes and long-term care facilities, a flexible yet structured approach is key. The choice of VR modality should be guided by the resident’s individual cognitive profile and tolerance. Partially immersive systems are ideal for group social and physical activities. such as a seated virtual cycling tour, which promotes light physical activity with minimal cybersickness risk. For residents requiring individualized stimulation, particularly those with apathy or agitation, supervised fully immersive VR can be offered. Here, the prescription is for experiences like exploring a “virtual aquarium,” which aims not at cognitive challenge but at providing sensory enrichment and emotional regulation, with staff monitoring tolerance closely.

In resource-limited environments, where advanced VR infrastructure is absent, the principles of precision immersion can still be applied by adapting available tools. Structured physical or cognitive training remains foundational. However, mobile VR (e.g., a smartphone in a low-cost cardboard headset) can be a scalable alternative for delivering immersive memory exercises. Furthermore, the core principle of cognitive-motor integration can be operationalized without any technology by designing simple, low-cost dual-task activities, such as walking while performing serial subtraction, which directly targets executive function and motor control.

This refined framework operationalizes the precision prescription model, providing concrete examples of how to match immersion level and task design to individual cognitive profiles and clinical environments. It shifts the paradigm from simply applying VR to thoughtfully prescribing it, thereby aligning with the broader biopsychosocial paradigm of mental health promotion through integrative physical–cognitive interventions.

### 4.5. Limitations and Future Directions

This study introduces the concept of “precision immersion-based intervention,” which advocates tailoring VR immersion levels and task complexity to individual cognitive phenotypes (e.g., memory vs. executive dysfunction) and sensory tolerances (e.g., visual load, motion sensitivity). This approach offers a conceptual foundation for future adaptive VR systems in cognitive rehabilitation.

Nonetheless, several limitations warrant consideration. First, although the analysis included outcomes related to psychological and functional well-being—namely the SGDS and IADL—no significant between-group differences were found. This may be attributed to limited statistical power from the small number of trials reporting these outcomes, or insufficient intervention duration to induce observable changes in affective or daily functioning. Furthermore, these domains are often influenced by broader psychosocial and environmental factors (e.g., caregiver support, lifestyle habits), which VR alone may not address effectively, especially in short-term RCTs.

Second, although the included studies varied in VR devices, content type, and intervention dose, this meta-analysis did not perform subgroup analyses based on these factors. The only subgroup comparison conducted was stratified by participant diagnosis, limiting conclusions regarding the relative contribution of specific intervention designs or hardware configurations. Additionally, non-immersive and hybrid VR systems were excluded to maintain methodological consistency and ensure a clear comparison between fully and partially immersive modalities. However, this decision may have introduced selection bias by omitting potentially relevant studies that utilized other technological approaches. Future research should broaden inclusion criteria and directly compare the full spectrum of VR immersion levels to determine the optimal combinations for different cognitive profiles.

To address this limitation, future studies should not only expand inclusion criteria but also conduct sensitivity analyses based on VR task type (e.g., cognitive, motor, or dual-task paradigms). Such analyses would help clarify whether different task compositions drive distinct neuroplastic and behavioral responses, providing deeper mechanistic insight into how VR engagement promotes cognitive recovery.

Third, long-term effects remain unclear, with only two studies including follow-up assessments ≥3 months. This limits our ability to evaluate the durability of VR-induced gains or their potential to influence disease progression and mental health maintenance over time (Li et al., 2025; J. G. Yang et al., 2022). Finally, the dose–response relationship for VR training is still poorly understood. Key parameters such as optimal frequency, session length, and cumulative training load are rarely reported or standardized, hindering cross-study comparisons and evidence-based clinical recommendations.

Fourth, the generalizability of our findings is constrained by the geographical and demographic characteristics of the available evidence. As noted in the results, the current literature is dominated by trials conducted in East Asia, with no eligible RCTs from North America or other Western populations. This geographical skew, combined with the prevalence of small-sample designs, poses a significant limitation to the external validity of our pooled results. Cultural factors, healthcare delivery systems, lifestyle habits, and genetic backgrounds can substantially influence both the implementation and effectiveness of non-pharmacological interventions. Therefore, caution is warranted when generalizing these findings to other ethnic and cultural contexts, and future multinational, large-scale trials are critically needed to validate and extend our conclusions.

Looking ahead, future trials should adopt multi-arm RCT designs directly comparing different VR modalities and immersion levels, while incorporating psychological, neuroimaging, and functional biomarkers to elucidate mechanisms of change. Integration of physical–cognitive dual-task training within VR—particularly for older adults—may enhance transfer to real-world functioning and promote mental well-being. Furthermore, AI-driven adaptive systems, capable of dynamically adjusting task complexity and immersion based on user performance represent a promising avenue for individualized and scalable digital rehabilitation.

Building upon the present findings and limitations, three pivotal directions emerge for future research. First, while this review employed comprehensive searches to mitigate publication bias, the body of primary literature remains susceptible to the under-reporting of negative results. Therefore, we strongly endorse the prospective registration and timely publication of all clinical trials as a foundational priority. Second, systematic investigation into the effects of cultural factors and rapid technological evolution is essential to determine the generalizability and real-world applicability of VR interventions across diverse populations and platforms. Third, and most critically, the establishment of long-term follow-up studies is imperative to validate the durability of cognitive and functional benefits and to assess the potential of VR to meaningfully alter the trajectory of cognitive decline.

## 5. Conclusions

This study confirms that the efficacy of VR as a cognitive intervention is not unilaterally dictated by technological complexity, but rather by the nuanced interplay between degree of immersion, cognitive domain impairment, and the activation of specific neural circuits. Fully immersive VR exhibits distinct advantages in enhancing spatial memory and cognitive arousal, likely through activation of the hippocampal–entorhinal circuit to optimize episodic memory encoding. In contrast, partially immersive VR consistently ranked highest across several core outcomes and appears particularly effective for improving executive function, attentional control, and emotional regulation, potentially via the prefrontal–striatal pathways.

These findings mark a shift in digital therapeutics—from a technology-centric paradigm to an era of “precision immersion prescriptions.” By tailoring VR modalities based on intervention goals, patient characteristics, and resource accessibility, we move closer to transforming VR from a generic training tool into a targeted rehabilitative strategy. In the context of accelerated global population aging, such individualized interventions are not only technological advances but also strategic imperatives for achieving international dementia prevention and management goals (Urbina, 2024).

In light of the accelerating global dementia prevalence and widespread cognitive decline among older adults, scalable and individualized VR-based interventions are not just technological innovations, but critical components of future public health strategy. Looking ahead, biomarker-guided adaptive VR systems—capable of modulating immersion and cognitive load in real time—may help realize the vision of precision digital medicine: delivering the right immersive experience, at the right time, to the right patient.

## Figures and Tables

**Table 1:** Basic Characteristics of Included Studies (N = 20).

Study (Year)	Disease Type	Sample Size (ET/CT)	Population Source	Age (Years, Mean ± SD)	Female (%)	Education (Years, Mean ± SD)	Intervention Cycle and Frequency
Li et al. (2025)	MCI & Dementia	232 (116/116)	Rural day care centers (China)	ET: 73.0 ± 4.4 CT: 72.7 ± 5.0	ET: 58.6 CT: 53.4	ET: 6.5 ± 3.0 CT: 6.3 ± 3.0	60 min/session, 2×/week, 12 weeks
Kwan et al. (2024)	Cognitive Frailty	293 (146/147)	Community centers (Hong Kong, China)	ET: 75.2 ± 7.1 CT: 73.9 ± 6.6	ET: 75.3 CT: 81.0	-	60 min/session, 2×/week, 8 weeks
Buele et al. (2024)	MCI	34 (17/17)	Community center (Ecuador)	ET: 75.4 ± 5.8 CT: 77.4 ± 6.8	ET: 58.8 CT: 76.5	ET: 5.5 ± 3.2 CT: 4.8 ± 3.3	40 min/session, 2×/week, 6 weeks
Chiu et al. (2023)	Cognitive Impairment	60 (30/30)	Long-term care facilities (Taiwan, China)	ET: 80.7 ± 8.8 CT: 80.0 ± 7.9	ET: 46.7 CT: 66.7	-	60 min/session, 1×/week, 8 weeks
Arshad et al. (2023)	MCI	51 (26/25)	Hospital rehabilitation unit (Pakistan)	ET: 62.9 ± 5.6 CT: 63.2 ± 5.1	ET: 61.5 CT: 60.0	-	30 min/session, 5×/week, 6 weeks
J. G. Yang et al. (2022)	MCI	99 (33/33/33) *	Community cohort (South Korea)	ET1: 72.5 ± 5.0 ET2: 67.9 ± 3.6 CT: 72.6 ± 5.6	ET1: 60.6 ET2: 90.9 CT: 81.8	ET1: 9.5 ± 3.7 ET2: 8.5 ± 3.9 CT: 8.5 ± 3.6	100 min/session, 3×/week, 8 weeks
J.-H. Park (2022)	MCI	32 (16/16)	Senior center (South Korea)	ET: 72.3 ± 5.1 CT: 70.9 ± 4.5	ET: 43.8 CT: 62.5	ET: 7.6 ± 3.9 CT: 7.5 ± 2.9	16 sessions, 2×/week, 8 weeks
Oliveira et al. (2021)	AD	17 (10/7)	Nursing home (Portugal)	ET: 82.6 ± 5.4 CT: 84.1 ± 6.3	ET: 70.0 CT: 71.4	-	45 min/session, 2×/week, 5 weeks
Kwan et al. (2021)	MCI	17 (9/8)	Community center (Hong Kong)	ET: 73.0 ± 5.6 CT: 77.5 ± 11.3	ET: 88.9 CT: 87.5	-	30 min/session, 2×/week, 8 weeks
Kang et al. (2021)	Prodromal Dementia	41 (23/18)	Memory clinic (South Korea)	ET: 75.5 ± 4.7 CT: 73.3 ± 7.0	ET: 73.9 CT: 66.7	ET: 7.7 ± 4.1 CT: 8.6 ± 4.8	20–30 min/session, 2×/week, 4 weeks
Thapa et al. (2020)	MCI	68 (34/34)	Regional healthcare centers (South Korea)	ET: 72.6 ± 5.4 CT: 72.7 ± 5.6	ET: 17.6 CT: 29.4	ET: 9.3 ± 4.0 CT: 8.4 ± 3.5	100 min/session, 3×/week, 8 weeks
J. S. Park et al. (2020)	MCI	34 (18/17)	Community-dwelling (South Korea)	ET: 75.8 ± 8.5 CT: 77.2 ± 7.2	ET: 55.6 CT: 58.8	-	30 min/session, 5×/week, 6 weeks
J.-H. Park et al. (2020)	aMCI	21 (10/11)	Hospital memory clinic (South Korea)	ET: 71.8 ± 6.6 CT: 69.5 ± 7.5	ET: 70.0 CT: 63.6	ET: 7.2 ± 3.6 CT: 8.0 ± 2.9	30 min/session, 2×/week, 12 weeks
Liao et al. (2020)	MCI	34 (18/16)	Day care centers (Taiwan, China)	ET: 75.5 ± 5.2 CT: 73.1 ± 6.8	ET: 61.1 CT: 75.0	ET: 9.3 ± 3.8 CT: 9.9 ± 2.1	60 min/session, 3×/week, 12 weeks
H.-L. Yang et al. (2019)	MCI	66 (33/33)	Retirement communities (Taiwan, China)	ET: 75.4 ± 6.6 CT: 81.7 ± 7.2	ET: 75.8 CT: 81.8	-	45 min/session, 3×/week, 12 weeks
Liao et al. (2019)	MCI	34 (18/16)	Community centers (Taiwan, China)	ET: 75.5 ± 5.2 CT: 73.1 ± 6.8	ET: 61.1 CT: 75.0	ET: 9.3 ± 3.8 CT: 9.9 ± 2.1	60 min/session, 3×/week, 12 weeks
Choi and Lee (2019)	MCI	60 (30/30)	Welfare center (South Korea)	ET: 77.3 ± 4.4 CT: 75.4 ± 4.0	ET: 83.3 CT: 86.7	-	60 min/session, 2×/week, 6 weeks
Delbroek et al. (2017)	MCI	20 (10/10)	Nursing home (Belgium)	ET: 86.9 ± 5.6 CT: 87.5 ± 6.6	ET: 80.0 CT: 50.0	-	18–30 min/session, 2×/week, 6 weeks
Serino et al. (2017)	AD	20 (10/10)	Social center (Italy)	ET: 86.6 ± 6.1 CT: 88.7 ± 3.6	ET: 90.0 CT: 80.0	ET: 9.8 ± 4.0 CT: 7.0 ± 5.0	20 min/session, 3×/week, 3–4 weeks
Baldimtsi et al. (2023)	MCI	67 (28/28/11) *	Day care center (Greece)	ET1: 66.1 ± 10.0 ET2: 73.0 ± 8.5 CT: 74.4 ± 7.0	ET1: 74.1 ET2: 100 CT: 85.7	-	32 sessions, 2–3×/week, 12 weeks

Notes: ET = Experimental group; CT = Control group; aMCI = Amnestic Mild Cognitive Impairment; * = Multi -arm trial; - = Data not reported; Cognitive Frailty = Coexistence of physical frailty and mild cognitive impairment; Cognitive Impairment = Broad term encompassing mild cognitive impairment, dementia, or other cognitive decline without specific subtype diagnosis.

## Data Availability

Some or all data generated or analyzed during this study are included in this published article or in the data repositories listed in References.

## Supplementary Materials

The following supporting information can be downloaded at https://www.mdpi.com/article/10.3390/bs15121610/s1.
