# Supplementary material for: Virtual Reality-Based Cognitive and Physical Interventions in Cognitive Impairment: A Network Meta-Analysis of Immersion Level Effects
Source: Behav Sci (Basel). 2025 Nov 22;15(12):1610. doi: 10.3390/bs15121610 (PMC12730044; doi:10.3390/bs15121610)
Supplement: Supplementary file 1 [file behavsci-15-01610-s001.zip › Supplement Search strategy.pdf]

PubMed Search Strategy

Search: (((("Cognitive Dysfunction"[Mesh]) OR ((((((((((((((((((((((((((Cognitive Dysfunctions[Title/Abstract]) OR (Dysfunction, Cognitive[Title/Abstract])) OR (Dysfunctions, Cognitive[Title/Abstract])) OR (Cognitive Disorder[Title/Abstract])) OR (Cognitive Disorders[Title/Abstract])) OR (Disorder, Cognitive[Title/Abstract])) OR (Disorders, Cognitive[Title/Abstract])) OR (Cognitive Impairments[Title/Abstract])) OR (Cognitive Impairment[Title/Abstract])) OR (Impairment, Cognitive[Title/Abstract])) OR (Impairments, Cognitive[Title/Abstract])) OR (Mild Cognitive Impairment[Title/Abstract])) OR (Cognitive Impairment, Mild[Title/Abstract])) OR (Cognitive Impairments, Mild[Title/Abstract])) OR (Impairment, Mild Cognitive[Title/Abstract])) OR (Impairments, Mild Cognitive[Title/Abstract])) OR (Mild Cognitive Impairments[Title/Abstract])) OR (Cognitive Decline[Title/Abstract])) OR (Cognitive Declines[Title/Abstract])) OR (Decline, Cognitive[Title/Abstract])) OR (Declines, Cognitive[Title/Abstract])) OR (Mental Deterioration[Title/Abstract])) OR (Deterioration, Mental[Title/Abstract])) OR (Deteriorations, Mental[Title/Abstract])) OR (Mental Deteriorations[Title/Abstract]))) OR (("Alzheimer Disease"[Mesh]) OR ((((((((((((((((((((((((((((((((((((Alzheimer Syndrome[Title/Abstract]) OR (Alzheimer-Type Dementia (ATD[Title/Abstract]))) OR (Alzheimer Type Dementia (ATD[Title/Abstract]))) OR (Dementia, Alzheimer-Type (ATD[Title/Abstract]))) OR (Alzheimer's Diseases[Title/Abstract])) OR (Alzheimer Diseases[Title/Abstract])) OR (Alzheimers Diseases[Title/Abstract])) OR (Alzheimer Dementia[Title/Abstract])) OR (Alzheimer Dementias[Title/Abstract])) OR (Dementia, Alzheimer[Title/Abstract])) OR (Alzheimer's Disease[Title/Abstract])) OR (Dementia, Senile[Title/Abstract])) OR (Senile Dementia[Title/Abstract])) OR (Dementia, Alzheimer Type[Title/Abstract])) OR (Alzheimer Type Dementia[Title/Abstract])) OR (Senile Dementia, Alzheimer Type[Title/Abstract])) OR (Alzheimer Type Senile Dementia[Title/Abstract])) OR (Primary Senile Degenerative Dementia[Title/Abstract])) OR (Alzheimer Sclerosis[Title/Abstract])) OR (Sclerosis, Alzheimer[Title/Abstract])) OR (Dementia, Primary Senile Degenerative[Title/Abstract])) OR (Dementia, Presenile[Title/Abstract])) OR (Presenile Dementia[Title/Abstract])) OR (Acute Confusional Senile Dementia[Title/Abstract])) OR (Senile Dementia, Acute Confusional[Title/Abstract])) OR (Alzheimer Disease, Early Onset[Title/Abstract])) OR (Early Onset Alzheimer Disease[Title/Abstract])) OR (Presenile Alzheimer Dementia[Title/Abstract])) OR (Alzheimer Disease, Late Onset[Title/Abstract])) OR (Late Onset Alzheimer Disease[Title/Abstract])) OR (Alzheimer's Disease, Focal Onset[Title/Abstract])) OR (Focal Onset Alzheimer's Disease[Title/Abstract])) OR (Familial Alzheimer Disease (FAD[Title/Abstract]))) OR (Alzheimer Disease, Familial (FAD[Title/Abstract]))) OR (Familial Alzheimer Diseases (FAD[Title/Abstract])))))) AND ((("Virtual Reality"[Mesh]) OR (((((((((((((Reality, Virtual[Title/Abstract]) OR (Virtual Reality, Educational[Title/Abstract])) OR (Educational Virtual Realities[Title/Abstract])) OR (Educational Virtual Reality[Title/Abstract])) OR (Reality, Educational Virtual[Title/Abstract])) OR (Virtual Realities, Educational[Title/Abstract])) OR (Virtual Reality, Instructional[Title/Abstract])) OR (Instructional Virtual Realities[Title/Abstract])) OR (Instructional Virtual Reality[Title/Abstract])) OR (Realities, Instructional Virtual[Title/Abstract])) OR (Reality, Instructional Virtual[Title/Abstract])) OR (Virtual Realities, Instructional[Title/Abstract]))) OR (("Virtual Reality Exposure Therapy"[Mesh]) OR ((((((((((((((((((((Virtual Reality Immersion Therapy[Title/Abstract]) OR (Virtual Reality Therapy[Title/Abstract])) OR (Reality Therapies,

Virtual[Title/Abstract])) OR (Reality Therapy, Virtual[Title/Abstract])) OR (Therapies, Virtual Reality[Title/Abstract])) OR (Therapy, Virtual Reality[Title/Abstract])) OR (Virtual Reality Therapies[Title/Abstract])) OR (Exergaming[Title/Abstract])) OR (Exergaming [Title/Abstract])) OR (Virtual Reality Exercise[Title/Abstract])) OR (Exercises, Virtual Reality[Title/Abstract])) OR (Exercise, Virtual Reality[Title/Abstract])) OR (Virtual Reality Exercises[Title/Abstract])) OR (Active-Video Gaming[Title/Abstract])) OR (Active Video Gaming[Title/Abstract])) OR (Active-Video Gamings[Title/Abstract])) OR (Gaming, Active-Video[Title/Abstract])) OR (Gamings, Active-Video[Title/Abstract])) OR (Exergames[Title/Abstract])) OR (Exergame[Title/Abstract]))))) AND (Randomized Controlled Trial [Publication Type] OR randomized [Title/Abstract] OR placebo [Title/Abstract])

Cochrane library

(Cognitive Dysfunctions):ab,ti,kw OR (Dysfunction, Cognitive):ab,ti,kw OR (Dysfunctions, Cognitive):ab,ti,kw OR (Cognitive Disorder):ab,ti,kw OR (Cognitive Disorders):ab,ti,kw OR (Disorder, Cognitive):ab,ti,kw OR (Disorders, Cognitive):ab,ti,kw OR (Cognitive Impairments):ab,ti,kw OR (Cognitive Impairment):ab,ti,kw OR (Impairment, Cognitive):ab,ti,kw OR (Impairments, Cognitive):ab,ti,kw OR (Mild Cognitive Impairment):ab,ti,kw OR (Cognitive Impairment, Mild):ab,ti,kw OR (Cognitive Impairments, Mild):ab,ti,kw OR (Impairment, Mild Cognitive):ab,ti,kw OR (Impairments, Mild Cognitive):ab,ti,kw OR (Mild Cognitive Impairments):ab,ti,kw OR (Cognitive Decline):ab,ti,kw OR (Cognitive Declines):ab,ti,kw OR (Decline, Cognitive):ab,ti,kw OR (Declines, Cognitive):ab,ti,kw OR (Mental Deterioration):ab,ti,kw OR (Deterioration, Mental):ab,ti,kw OR (Deteriorations, Mental):ab,ti,kw OR (Mental Deteriorations):ab,ti,kw OR (Alzheimer Syndrome):ab,ti,kw OR (Alzheimer-Type Dementia (ATD)):ab,ti,kw OR (Alzheimer Type Dementia (ATD)):ab,ti,kw OR (Dementia, Alzheimer-Type (ATD)):ab,ti,kw OR (Alzheimer's Diseases):ab,ti,kw OR (Alzheimer Diseases):ab,ti,kw OR (Alzheimers Diseases):ab,ti,kw OR (Alzheimer Dementia):ab,ti,kw OR (Alzheimer Dementias):ab,ti,kw OR (Dementia, Alzheimer):ab,ti,kw OR (Alzheimer's Disease):ab,ti,kw OR (Dementia, Senile):ab,ti,kw OR (Senile Dementia):ab,ti,kw OR (Dementia, Alzheimer Type):ab,ti,kw OR (Alzheimer Type Dementia):ab,ti,kw OR (Senile Dementia, Alzheimer Type):ab,ti,kw OR (Alzheimer Type Senile Dementia):ab,ti,kw OR (Primary Senile Degenerative Dementia):ab,ti,kw OR (Alzheimer Sclerosis):ab,ti,kw OR (Sclerosis, Alzheimer):ab,ti,kw OR (Dementia, Primary Senile Degenerative):ab,ti,kw OR (Dementia, Presenile):ab,ti,kw OR (Presenile Dementia):ab,ti,kw OR (Acute Confusional Senile Dementia):ab,ti,kw OR (Senile Dementia, Acute Confusional):ab,ti,kw OR (Alzheimer Disease, Early Onset):ab,ti,kw OR (Early Onset Alzheimer Disease):ab,ti,kw OR (Presenile Alzheimer Dementia):ab,ti,kw OR (Alzheimer Disease, Late Onset):ab,ti,kw OR (Late Onset Alzheimer Disease):ab,ti,kw OR (Alzheimer's Disease, Focal Onset):ab,ti,kw OR (Focal Onset Alzheimer's Disease):ab,ti,kw OR (Familial Alzheimer Disease (FAD)):ab,ti,kw OR (Alzheimer Disease, Familial (FAD)):ab,ti,kw OR (Familial Alzheimer Diseases (FAD)):ab,ti,kw AND (Reality, Virtual):ab,ti,kw OR (Virtual Reality, Educational):ab,ti,kw OR (Educational Virtual Realities):ab,ti,kw OR (Educational Virtual Reality):ab,ti,kw OR (Reality, Educational Virtual):ab,ti,kw OR (Virtual Realities, Educational):ab,ti,kw OR (Virtual Reality, Instructional):ab,ti,kw OR (Instructional Virtual Realities):ab,ti,kw OR (Instructional Virtual Reality):ab,ti,kw OR (Realities, Instructional Virtual):ab,ti,kw OR (Reality, Instructional

Virtual):ab,ti,kw OR (Virtual Realities, Instructional):ab,ti,kw OR (Virtual Reality Exposure Therapy):ab,ti,kw OR (Virtual Reality Immersion Therapy):ab,ti,kw OR (Virtual Reality Therapy):ab,ti,kw OR (Reality Therapies, Virtual):ab,ti,kw OR (Reality Therapy, Virtual):ab,ti,kw OR (Therapies, Virtual Reality):ab,ti,kw OR (Therapy, Virtual Reality):ab,ti,kw OR (Virtual Reality Therapies):ab,ti,kw OR (Exergaming):ab,ti,kw OR (Exergamings):ab,ti,kw OR (Virtual Reality Exercise):ab,ti,kw OR (Exercises, Virtual Reality):ab,ti,kw OR (Exercise, Virtual Reality):ab,ti,kw OR (Virtual Reality Exercises):ab,ti,kw OR (Active-Video Gaming):ab,ti,kw OR (Active Video Gaming):ab,ti,kw OR (Active-Video Gamings):ab,ti,kw OR (Gaming, Active-Video):ab,ti,kw OR (Gamings, Active-Video):ab,ti,kw OR (Exergames):ab,ti,kw OR (Exergame):ab,ti,kw AND (Randomized Controlled Trial):ab,ti,kw OR (randomized):ab,ti,kw OR (placebo):ab,ti,kw OR (RCT):ab,ti,kw

Embase Search Strategy

'Cognitive Dysfunctions':ab,ti OR 'Dysfunction, Cognitive':ab,ti OR 'Dysfunctions, Cognitive':ab,ti OR 'Cognitive Disorder':ab,ti OR 'Cognitive Disorders':ab,ti OR 'Disorder, Cognitive':ab,ti OR 'Disorders, Cognitive':ab,ti OR 'Cognitive Impairments':ab,ti OR 'Cognitive Impairment':ab,ti OR 'Impairment, Cognitive':ab,ti OR 'Impairments, Cognitive':ab,ti OR 'Mild Cognitive Impairment':ab,ti OR 'Cognitive Impairment, Mild':ab,ti OR 'Cognitive Impairments, Mild':ab,ti OR 'Impairment, Mild Cognitive':ab,ti OR 'Impairments, Mild Cognitive':ab,ti OR 'Mild Cognitive Impairments':ab,ti OR 'Cognitive Decline':ab,ti OR 'Cognitive Declines':ab,ti OR 'Decline, Cognitive':ab,ti OR 'Declines, Cognitive':ab,ti OR 'Mental Deterioration':ab,ti OR 'Deterioration, Mental':ab,ti OR 'Deteriorations, Mental':ab,ti OR 'Mental Deteriorations':ab,ti OR 'Alzheimer Syndrome':ab,ti OR 'Alzheimer-Type Dementia (ATD)':ab,ti OR 'Alzheimer Type Dementia (ATD)':ab,ti OR 'Dementia, Alzheimer-Type (ATD)':ab,ti OR 'Alzheimer Diseases':ab,ti OR 'Alzheimer Diseases':ab,ti OR 'Alzheimers Diseases':ab,ti OR 'Alzheimer Dementia':ab,ti OR 'Alzheimer Dementias':ab,ti OR 'Dementia, Alzheimer':ab,ti OR 'Alzheimer Disease':ab,ti OR 'Dementia, Senile':ab,ti OR 'Senile Dementia':ab,ti OR 'Dementia, Alzheimer Type':ab,ti OR 'Alzheimer Type Dementia':ab,ti OR 'Senile Dementia, Alzheimer Type':ab,ti OR 'Alzheimer Type Senile Dementia':ab,ti OR 'Primary Senile Degenerative Dementia':ab,ti OR 'Alzheimer Sclerosis':ab,ti OR 'Sclerosis, Alzheimer':ab,ti OR 'Dementia, Primary Senile Degenerative':ab,ti OR 'Dementia, Presenile':ab,ti OR 'Presenile Dementia':ab,ti OR 'Acute Confusional Senile Dementia':ab,ti OR 'Senile Dementia, Acute Confusional':ab,ti OR 'Alzheimer Disease, Early Onset':ab,ti OR 'Early Onset Alzheimer Disease':ab,ti OR 'Presenile Alzheimer Dementia':ab,ti OR 'Alzheimer Disease, Late Onset':ab,ti OR 'Late Onset Alzheimer Disease':ab,ti OR 'Alzheimer Disease, Focal Onset':ab,ti OR 'Focal Onset Alzheimer Disease':ab,ti OR 'Familial Alzheimer Disease (FAD)':ab,ti OR 'Alzheimer Disease, Familial (FAD)':ab,ti OR 'Familial Alzheimer Diseases (FAD)':ab,ti AND 'Reality, Virtual':ab,ti OR 'Virtual Reality, Educational':ab,ti OR 'Educational Virtual Realities':ab,ti OR 'Educational Virtual Reality':ab,ti OR 'Reality, Educational Virtual':ab,ti OR 'Virtual Realities, Educational':ab,ti OR 'Virtual Reality, Instructional':ab,ti OR 'Instructional Virtual Realities':ab,ti OR 'Instructional Virtual Reality':ab,ti OR 'Realities, Instructional Virtual':ab,ti OR 'Reality, Instructional Virtual':ab,ti OR 'Virtual Realities, Instructional':ab,ti OR 'Virtual Reality Exposure Therapy':ab,ti OR 'Virtual Reality Immersion Therapy':ab,ti OR 'Virtual Reality Therapy':ab,ti OR 'Reality Therapies, Virtual':ab,ti OR 'Reality Therapy, Virtual':ab,ti OR 'Therapies, Virtual Reality':ab,ti OR 'Therapy, Virtual

Reality':ab,ti OR 'Virtual Reality Therapies':ab,ti OR 'Exergaming':ab,ti OR 'Exergamings':ab,ti OR 'Virtual Reality Exercise':ab,ti OR 'Exercises, Virtual Reality':ab,ti OR 'Exercise, Virtual Reality':ab,ti OR 'Virtual Reality Exercises':ab,ti OR 'Active-Video Gaming':ab,ti OR 'Active Video Gaming':ab,ti OR 'Active-Video Gamings':ab,ti OR 'Gaming, Active-Video':ab,ti OR 'Gamings, Active-Video':ab,ti OR 'Exergames':ab,ti OR 'Exergame':ab,ti AND 'Randomized Controlled Trial':ab,ti OR 'Randomized':ab,ti OR 'Placebo':ab,ti OR 'RCT':ab,ti

Web of science Search Strategy

Cognitive Dysfunction OR Cognitive Dysfunctions OR Dysfunction, Cognitive OR Dysfunctions, Cognitive OR Cognitive Disorder OR Cognitive Disorders OR Disorder, Cognitive OR Disorders, Cognitive OR Cognitive Impairments OR Cognitive Impairment OR Impairment, Cognitive OR Impairments, Cognitive OR Mild Cognitive Impairment OR Cognitive Impairment, Mild OR Cognitive Impairments, Mild OR Impairment, Mild Cognitive OR Impairments, Mild Cognitive OR Mild Cognitive Impairments OR Cognitive Decline OR Cognitive Declines OR Decline, Cognitive OR Declines, Cognitive OR Mental Deterioration OR Deterioration, Mental OR Deteriorations, Mental OR Mental Deteriorations OR Alzheimer Disease OR Alzheimer Syndrome OR Alzheimer-Type Dementia (ATD) OR Alzheimer Type Dementia (ATD) OR Dementia, Alzheimer-Type (ATD) OR Alzheimer's Diseases OR Alzheimer Diseases OR Alzheimers Diseases OR Alzheimer Dementia OR Alzheimer Dementias OR Dementia, Alzheimer OR Alzheimer's Disease OR Dementia, Senile OR Senile Dementia OR Dementia, Alzheimer Type OR Alzheimer Type Dementia OR Senile Dementia, Alzheimer Type OR Alzheimer Type Senile Dementia OR Primary Senile Degenerative Dementia OR Alzheimer Sclerosis OR Sclerosis, Alzheimer OR Dementia, Primary Senile Degenerative OR Dementia, Presenile OR Presenile Dementia OR Acute Confusional Senile Dementia OR Senile Dementia, Acute Confusional OR Alzheimer Disease, Early Onset OR Early Onset Alzheimer Disease OR Presenile Alzheimer Dementia OR Alzheimer Disease, Late Onset OR Late Onset Alzheimer Disease OR Alzheimer's Disease, Focal Onset OR Focal Onset Alzheimer's Disease OR Familial Alzheimer Disease (FAD) OR Alzheimer Disease, Familial (FAD) OR Familial Alzheimer Diseases (FAD) AND Virtual reality OR Reality, Virtual OR Virtual Reality, Educational OR Educational Virtual Realities OR Educational Virtual Reality OR Reality, Educational Virtual OR Virtual Realities, Educational OR Virtual Reality, Instructional OR Instructional Virtual Realities OR Instructional Virtual Reality OR Realities, Instructional Virtual OR Reality, Instructional Virtual OR Virtual Realities, Instructional OR Virtual Reality Exposure Therapy OR Cognitive Impairments, Mild OR Virtual Reality Immersion Therapy OR Virtual Reality Therapy OR Reality Therapies, Virtual OR Reality Therapy, Virtual OR Therapies, Virtual Reality OR Therapy, Virtual Reality OR Virtual Reality Therapies OR Exergaming OR Exergamings OR Virtual Reality Exercise OR Exercises, Virtual Reality OR Exercise, Virtual Reality OR Virtual Reality Exercises OR Active-Video Gaming OR Active Video Gaming OR Active-Video Gamings OR Gaming, Active-Video OR Gamings, Active-Video OR Exergames OR Exergame AND Randomized Controlled Trial OR Randomized OR Placebo OR RCT
