# Supplementary material for: Virtual Reality-Based Cognitive and Physical Interventions in Cognitive Impairment: A Network Meta-Analysis of Immersion Level Effects
Source: Behav Sci (Basel). 2025 Nov 22;15(12):1610. doi: 10.3390/bs15121610 (PMC12730044; doi:10.3390/bs15121610)
Supplement: Supplementary file 1 [file behavsci-15-01610-s001.zip › Supplementary figure.pdf]

Supplementary Figure

MOCA

(1)B vs C

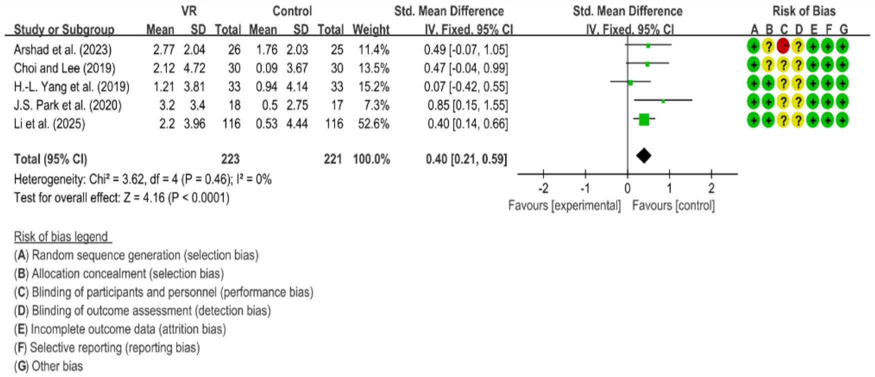

(2)A vs C

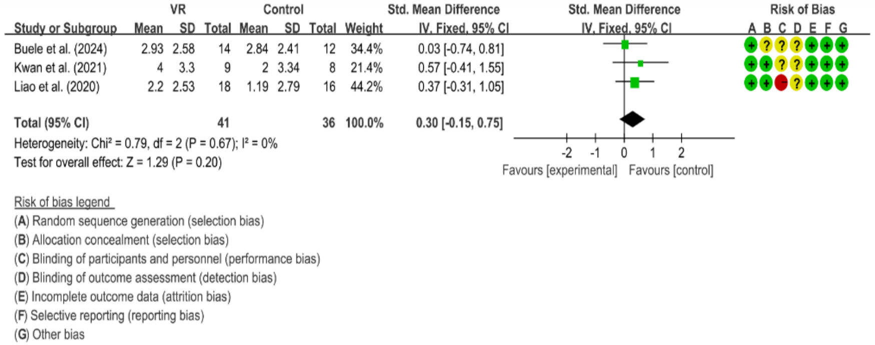

(3)A vs D

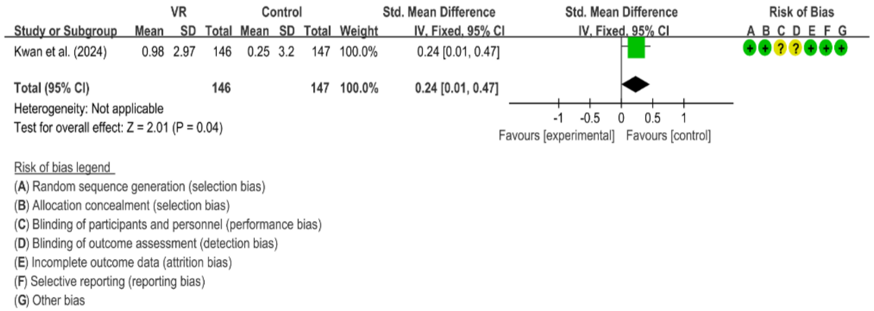

(4)B vs D

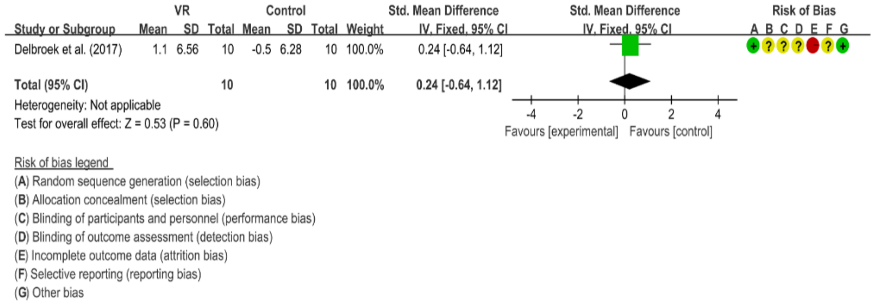

Figure S1. Forest plots of MOCA outcomes across intervention comparisons. (1) Comparison between Partially Immersive VR (B) and Active Control (C);(2) Comparison between Fully Immersive VR (A) and Active Control (C);(3) Comparison between Fully Immersive VR (A) and Passive Control (D);(4) Comparison between Partially Immersive VR (B) and Passive Control (D); Effect sizes are presented as standardized mean differences (SMDs) with 95% confidence intervals (CIs). A positive SMD indicates greater improvement in the VR or active intervention group compared to the control. The included studies are:(Arshad et al. (2023), Choi and Lee (2019), Park et al. (2020), J. S. Park et al. (2020), Li et al. (2025), Kwan et al. (2021), Liao et al. (2020), Kwan et al. (2024), Delbroek et al. (2017), and Buele et al. (2024)).

MOCA

A

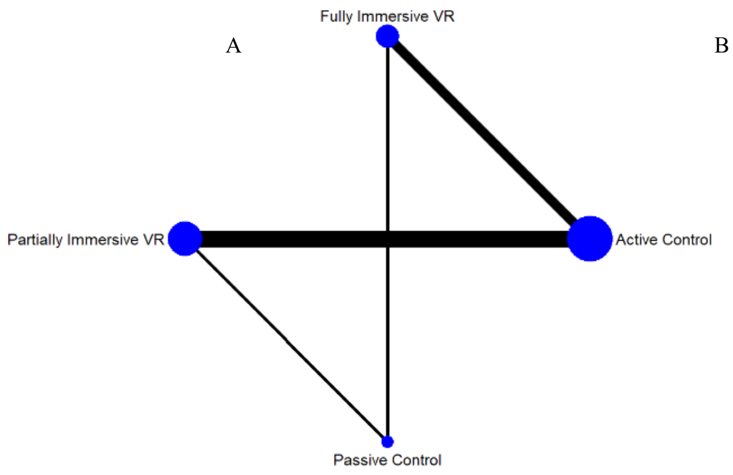

B

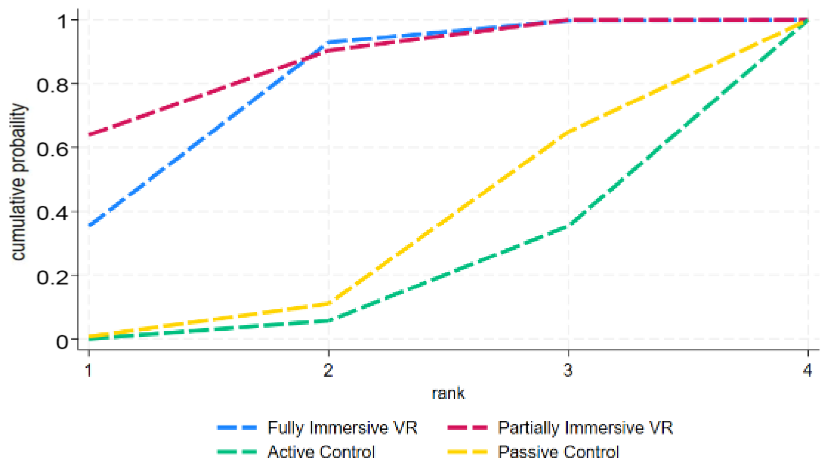

C

**Partially Immersive VR**			
**0.08 (−0.35,0.51)**	**Fully Immersive VR**		
**0.31 (−0.15,0.77)**	**0.23 (0.01,0.45)**	**Passive Control**	
**0.40 (0.21,0.58)**	**0.32 (−0.09,0.72)**	**0.09 (−0.36,0.53)**	**Active Control**

Figure S2. Network meta-analysis results of MOCA outcomes. (A) Network plot showing direct and indirect comparisons; node size reflects sample size, and edge thickness represents the number of comparisons. (B) SUCRA ranking plot of each intervention, where Surface Under the Cumulative Ranking Curve indicate better relative efficacy. (C) League table presenting the pairwise standardized mean differences (SMDs) and 95% confidence intervals (CIs) for all comparisons among interventions.

CASI

(1)A vs D

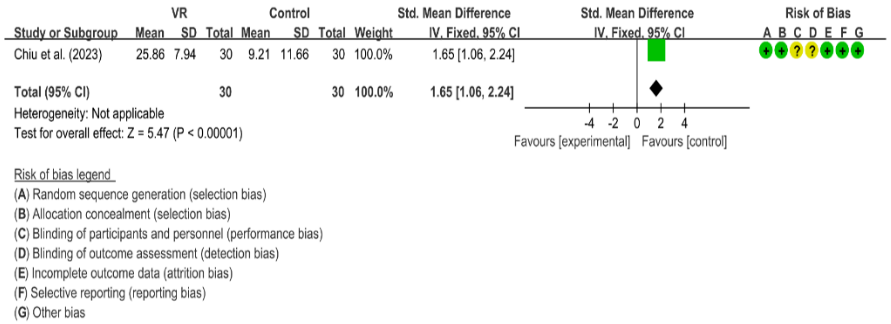

(2)B vs C

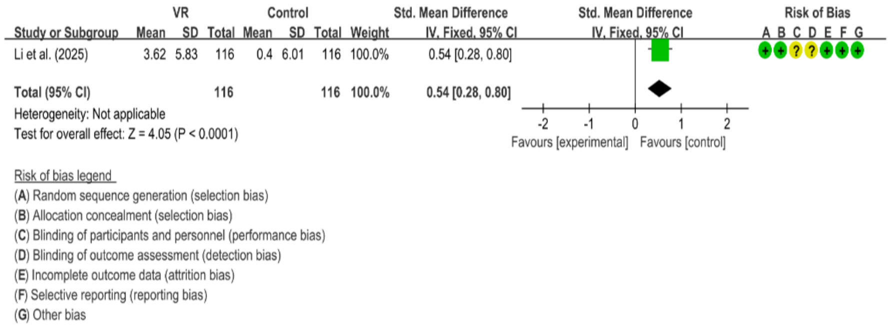

Figure S3. Forest plots of CASI outcomes across intervention comparisons. (1) Comparison between Fully Immersive VR (A) and Passive Control (D); (2) Comparison between Partially Immersive VR (B) and Active Control (C); Effect sizes are presented as standardized mean differences (SMDs) with 95% confidence intervals (CIs). A positive SMD indicates greater improvement in the VR or active intervention group compared to the control. The included studies are: (Chiu et al. (2023), Li et al. (2025)).

TMT-A

(1)A vs C

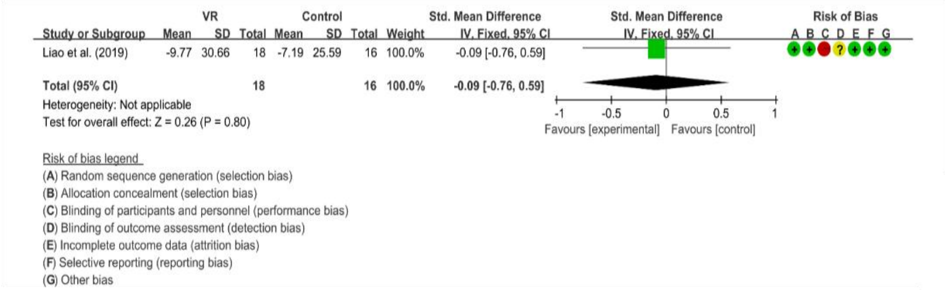

(2)A vs D

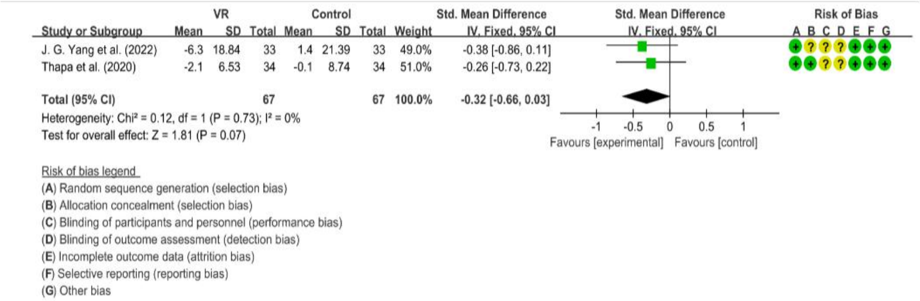

(3)B vs C

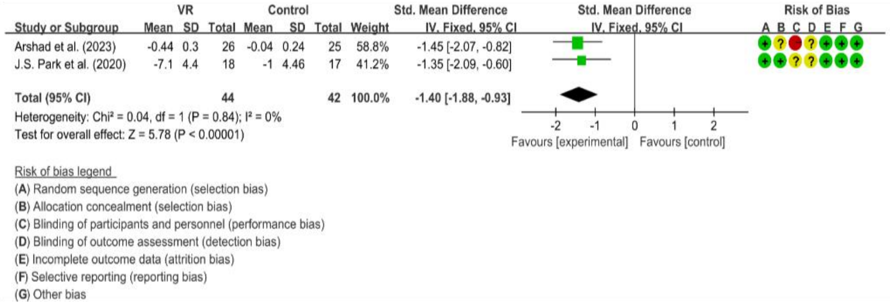

Figure S4. Forest plots of TMT-A outcomes across intervention comparisons. (1) Comparison between Fully Immersive VR (A) and Active Control (C);(2) Comparison between Fully Immersive VR (A) and Passive Control (D);(3) Comparison between Partially Immersive VR (B) and Active Control (C); Effect sizes are presented as standardized mean differences (SMDs) with 95% confidence intervals (CIs). A positive SMD indicates greater improvement in the VR or active intervention group compared to the control. The included studies are: The included studies are: (Liao et al. (2019), J. G. Yang et al. (2022), Thapa et al. (2020), Arshad et al. (2023), J. S. Park et al. (2020)).

TMT − A

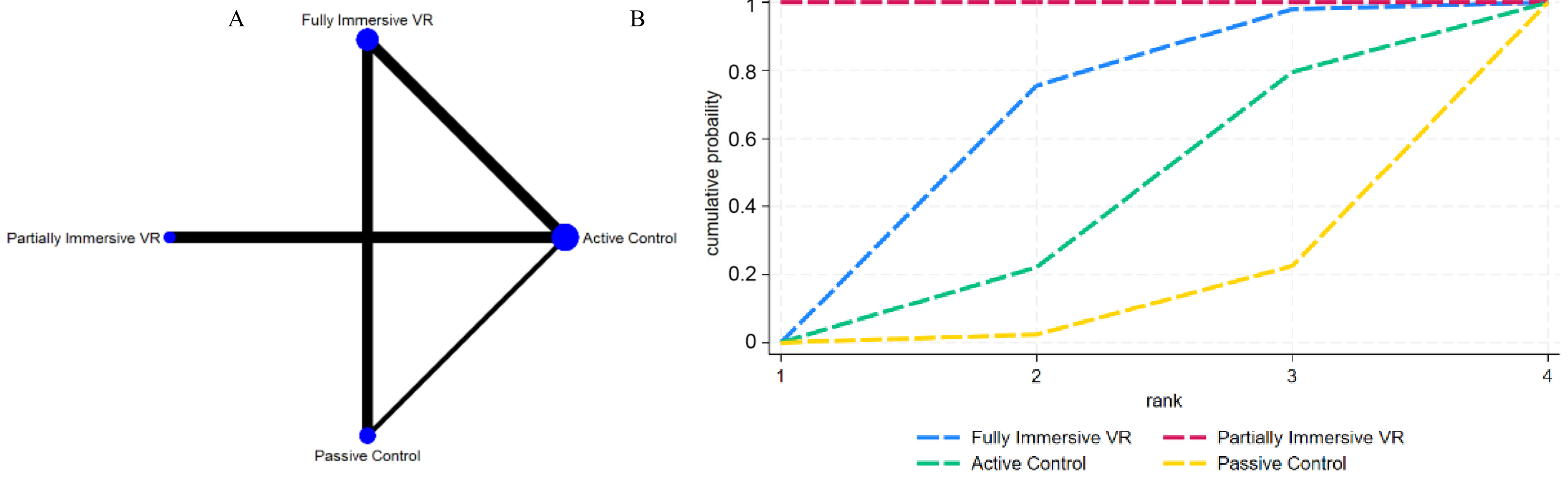

C

**Partially Immersive VR**			
**−1.26 (−1.87,−0.65)**	**Fully Immersive VR**		
**−1.40 (−1.88,−0.93)**	**−0.14 (−0.52,0.23)**	**Active Control**	
**−1.57 (−2.21,−0.94)**	**−0.31 (−0.65,0.02)**	**−0.17 (−0.58,0.24)**	**Passive Control**

Figure S5. Network meta-analysis results of TMT-A outcomes. (A) Network plot showing direct and indirect comparisons; node size reflects sample size, and edge thickness represents the number of comparisons. (B) SUCRA ranking plot of each intervention, where Surface Under the Cumulative Ranking Curve indicate better relative efficacy. (C) League table presenting the pairwise standardized mean differences (SMDs) and 95% confidence intervals (CIs) for all comparisons among interventions.

TMT-B

(1)A vs C

Study or Subgroup	VR Mean	VR SD	VR Total	Control Mean	Control SD	Control Total	Weight	Std. Mean Difference IV, Fixed, 95% CI
Liao et al. (2019)	-45.01	53.82	18	-18.13	57.51	16	100.0%	-0.47 [-1.16, 0.21]
**Total (95% CI)**			**18**			**16**	**100.0%**	**-0.47 [-1.16, 0.21]**

Heterogeneity: Not applicable
Test for overall effect: Z = 1.35 (P = 0.18)

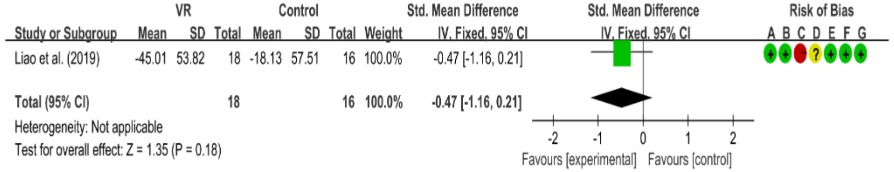

Risk of bias legend
(A) Random sequence generation (selection bias)
(B) Allocation concealment (selection bias)
(C) Blinding of participants and personnel (performance bias)
(D) Blinding of outcome assessment (detection bias)
(E) Incomplete outcome data (attrition bias)
(F) Selective reporting (reporting bias)
(G) Other bias

(2)A vs D

Study or Subgroup	VR Mean	VR SD	VR Total	Control Mean	Control SD	Control Total	Weight	Std. Mean Difference IV, Fixed, 95% CI
Baldimtsi et al. (2023)	-16.81	47.31	28	20.18	72.54	28	44.5%	-0.60 [-1.13, -0.06]
Thapa et al. (2020)	-5.3	24.9	34	4.7	26.73	34	55.5%	-0.38 [-0.86, 0.10]
**Total (95% CI)**			**62**			**62**	**100.0%**	**-0.48 [-0.84, -0.12]**

Heterogeneity: Chi² = 0.34, df = 1 (P = 0.56); I² = 0%
Test for overall effect: Z = 2.62 (P = 0.009)

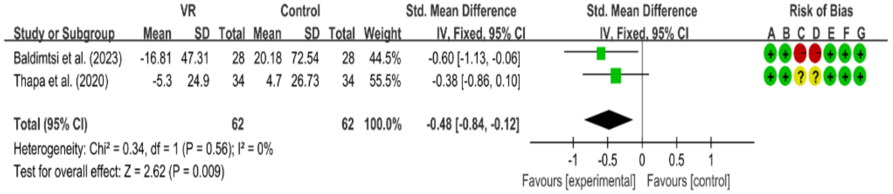

Risk of bias legend
(A) Random sequence generation (selection bias)
(B) Allocation concealment (selection bias)
(C) Blinding of participants and personnel (performance bias)
(D) Blinding of outcome assessment (detection bias)
(E) Incomplete outcome data (attrition bias)
(F) Selective reporting (reporting bias)
(G) Other bias

(3)B vs C

Study or Subgroup	VR Mean	VR SD	VR Total	Control Mean	Control SD	Control Total	Weight	Std. Mean Difference IV, Random, 95% CI
Arshad et al. (2023)	-1.26	0.53	26	-0.12	0.61	25	50.0%	-1.97 [-2.64, -1.29]
J.S. Park et al. (2020)	-7.9	8.49	18	-1.7	11.05	17	50.0%	-0.62 [-1.30, 0.06]
**Total (95% CI)**			**44**			**42**	**100.0%**	**-1.29 [-2.62, 0.03]**

Heterogeneity: Tau² = 0.79; Chi² = 7.59, df = 1 (P = 0.006); I² = 87%
Test for overall effect: Z = 1.91 (P = 0.06)

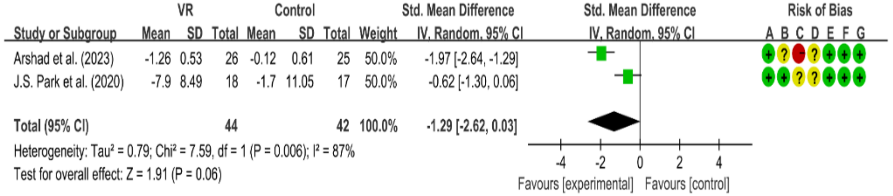

Risk of bias legend
(A) Random sequence generation (selection bias)
(B) Allocation concealment (selection bias)
(C) Blinding of participants and personnel (performance bias)
(D) Blinding of outcome assessment (detection bias)
(E) Incomplete outcome data (attrition bias)
(F) Selective reporting (reporting bias)
(G) Other bias

Figure S6. Forest plots of TMT-B outcomes across intervention comparisons. (1) Comparison between Fully Immersive VR (A) and Active Control (C);(2) Comparison between Fully Immersive VR (A) and Passive Control (D);(3) Comparison between Partially Immersive VR (B) and Active Control (C); Effect sizes are presented as standardized mean differences (SMDs) with 95% confidence intervals (CIs). A positive SMD indicates greater improvement in the VR or active intervention group compared to the control. The included studies are: (Liao et al. (2019), Arshad et al. (2023), Thapa et al. (2020), J. S. Park et al. (2020), Baldimtsi et al. (2023))

TMT − B

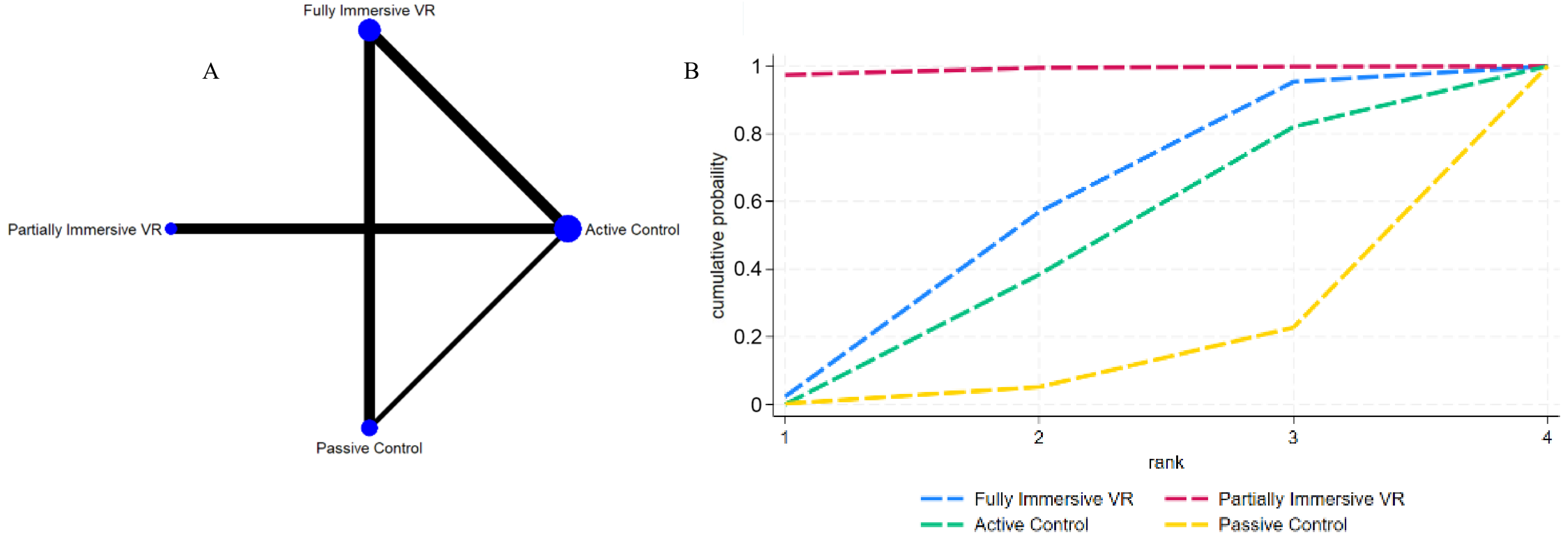

C

**Partially Immersive VR**			
**−1.18 (−2.37,0.01)**	**Fully Immersive VR**		
**−1.29 (−2.14,−0.44)**	**−0.11 (−0.94,0.72)**	**Active Control**	
**−1.74 (−3.03,−0.45)**	**−0.56 (−1.33,0.20)**	**−0.45 (−1.43,0.53)**	**Passive Control**

Figure S7. Network meta-analysis results of TMT-B outcomes. (A) Network plot showing direct and indirect comparisons; node size reflects sample size, and edge thickness represents the number of comparisons. (B) SUCRA ranking plot of each intervention, where Surface Under the Cumulative Ranking Curve indicate better relative efficacy. (C) League table presenting the pairwise standardized mean differences (SMDs) and 95% confidence intervals (CIs) for all comparisons among interventions.

SDST

(1)A vs D

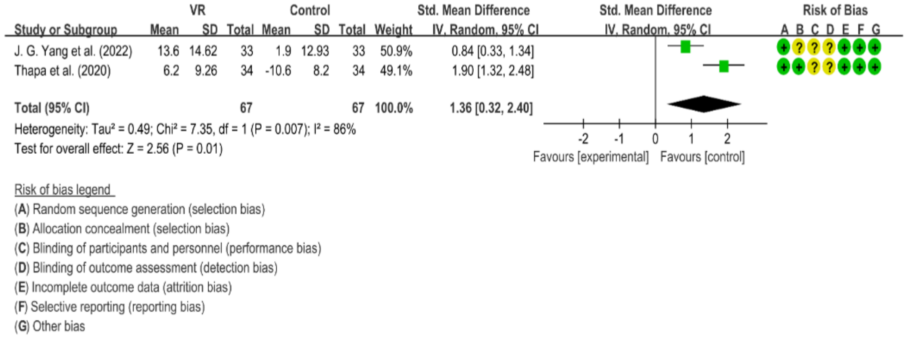

Figure S8. Forest plots of SDST outcomes across intervention comparisons. (1) Comparison between Fully Immersive VR (A) and Passive Control (D); Effect sizes are presented as standardized mean differences (SMDs) with 95% confidence intervals (CIs). A positive SMD indicates greater improvement in the VR or active intervention group compared to the control. The included studies are: (J. G. Yang et al. (2022), Thapa et al. (2020)).

DST-forward

(1)A vs D

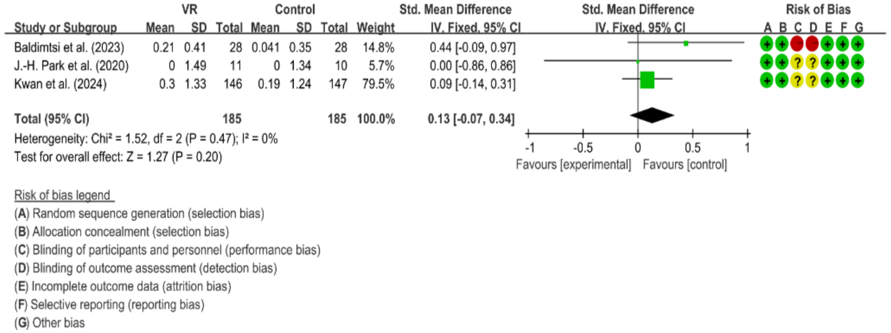

(2)B vs C

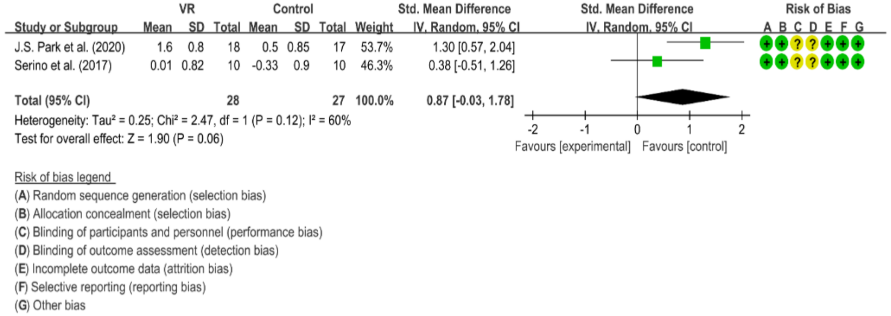

Figure S9. Forest plots of DST-forward outcomes across intervention comparisons (1) Comparison between Fully Immersive VR (A) and Passive Control (D); (2) Comparison between Partially Immersive VR (B) and Active Control (C); Effect sizes are presented as standardized mean differences (SMDs) with 95% confidence intervals (CIs). A positive SMD indicates greater improvement in the VR or active intervention group compared to the control. The included studies are: (Baldimtsi et al. (2023), J.-H. Park et al. (2020), Kwan et al. (2024), J. S. Park et al. (2020), Serino et al. (2017)).

DST − forward

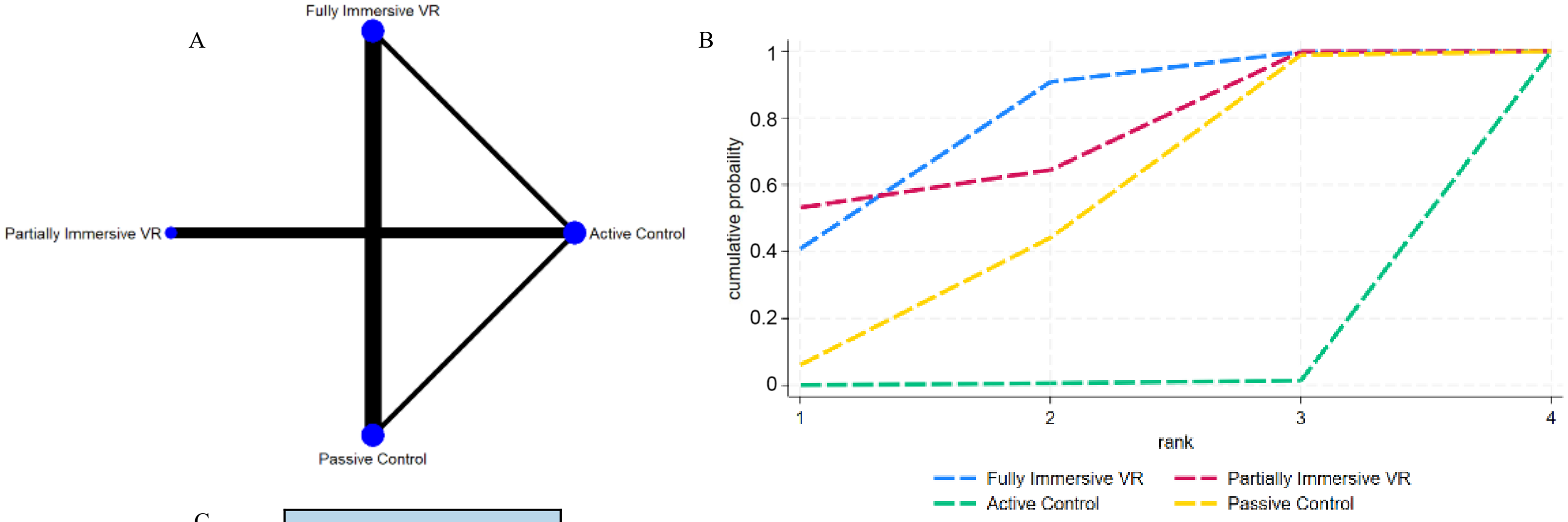

C

**Partially Immersive VR**			
**0.05 (−0.83,0.93)**	**Fully Immersive VR**		
**0.16 (−0.71,1.04)**	**0.11 (−0.09,0.31)**	**Passive Control**	
**0.92 (0.35,1.49)**	**0.87 (0.20,1.54)**	**0.76 (0.09,1.43)**	**Active Control**

Figure S10. Network meta-analysis results of DST-forward outcomes. (A) Network plot showing direct and indirect comparisons; node size reflects sample size, and edge thickness represents the number of comparisons. (B) SUCRA ranking plot of each intervention, where Surface Under the Cumulative Ranking Curve indicate better relative efficacy. (C) League table presenting the pairwise standardized mean differences (SMDs) and 95% confidence intervals (CIs) for all comparisons among interventions.

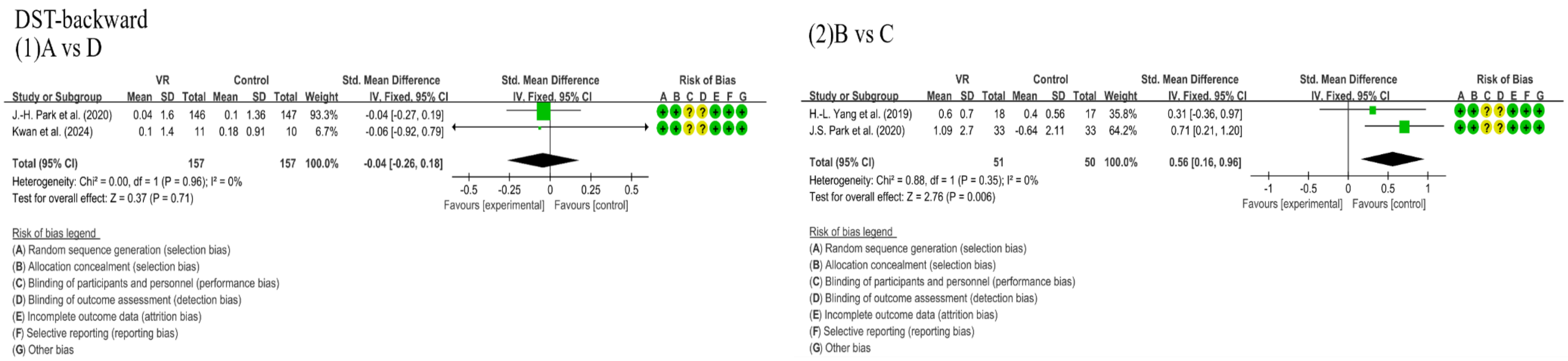

Figure S11. Forest plots of DST-backward outcomes across intervention comparisons. (1) Comparison between Fully Immersive VR (A) and Passive Control (D); (2) Comparison between Partially Immersive VR (B) and Active Control (C); Effect sizes are presented as standardized mean differences (SMDs) with 95% confidence intervals (CIs). A positive SMD indicates greater improvement in the VR or active intervention group compared to the control. The included studies are: (J.-H. Park et al. (2020), Kwan et al. (2024), H.-L. Yang et al. (2019), J. S. Park et al. (2020)).

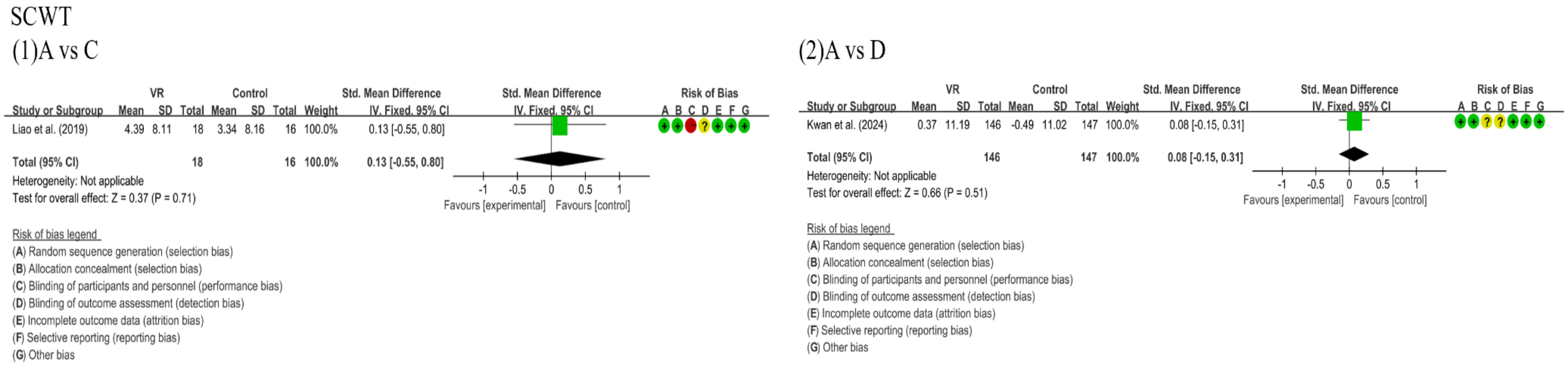

Figure S12. Forest plots of SCWT outcomes across intervention comparisons. (1) Comparison between Fully Immersive VR (A) and Active Control (C); (2) Comparison between Fully Immersive VR (A) and Passive Control (D); Effect sizes are presented as standardized mean differences (SMDs) with 95% confidence intervals (CIs). A positive SMD indicates greater improvement in the VR or active intervention group compared to the control. The included studies are: (Liao et al. (2019), Kwan et al. (2024)).

VFT

(1)A vs D

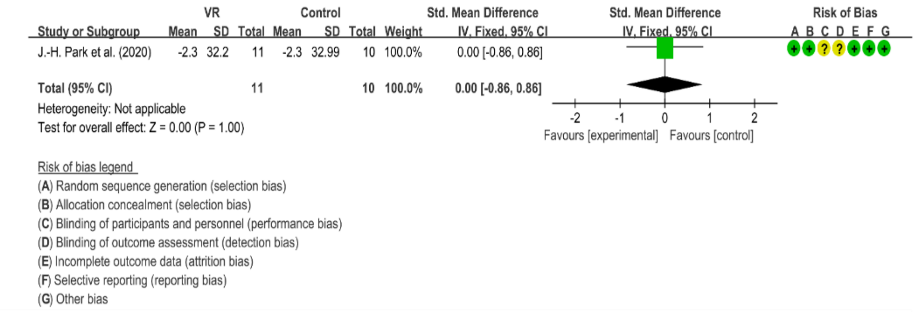

(2)B vs C

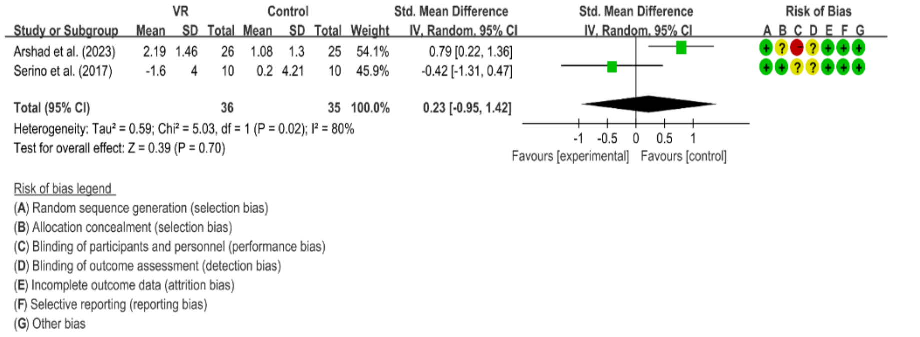

Figure S13. Forest plots of VFT outcomes across intervention comparisons. (1) Comparison between Fully Immersive VR (A) and Passive Control (D); (2) Comparison between Partially Immersive VR (B) and Active Control (C); Effect sizes are presented as standardized mean differences (SMDs) with 95% confidence intervals (CIs). A positive SMD indicates greater improvement in the VR or active intervention group compared to the control. The included studies are: (J.-H. Park et al. (2020), Arshad et al. (2023), Serino et al. (2017)).

FAB

(1)B vs C

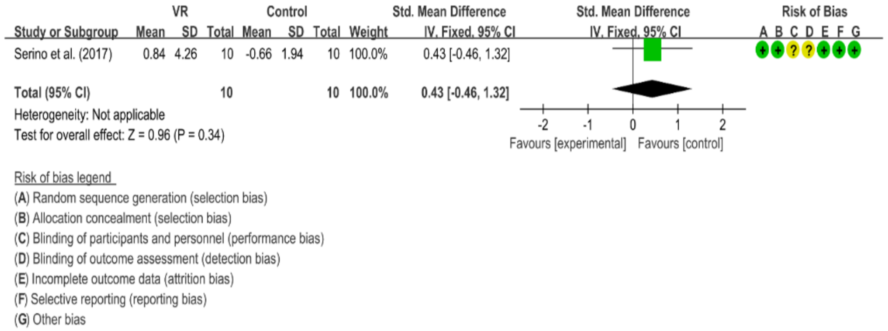

(2)B vs D

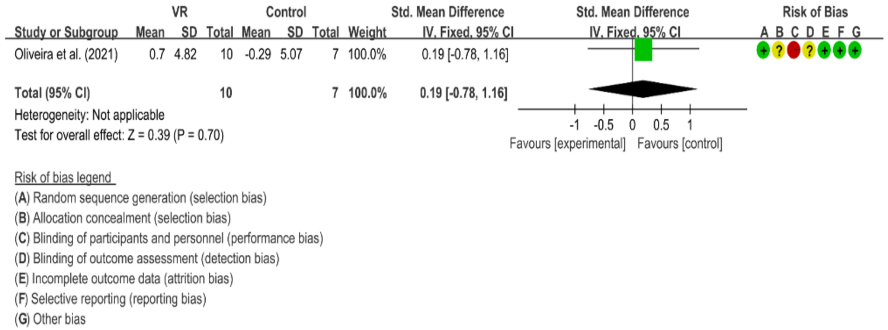

Figure S14. Forest plots of FAB outcomes across intervention comparisons. (1) Comparison between Partially Immersive VR (B) and Active Control (C); (2) Comparison between Partially Immersive VR (B) and Passive Control (D); Effect sizes are presented as standardized mean differences (SMDs) with 95% confidence intervals (CIs). A positive SMD indicates greater improvement in the VR or active intervention group compared to the control. The included studies are: (Serino et al. (2017), Oliveira et al. (2021)).

TUG

(1)A vs C

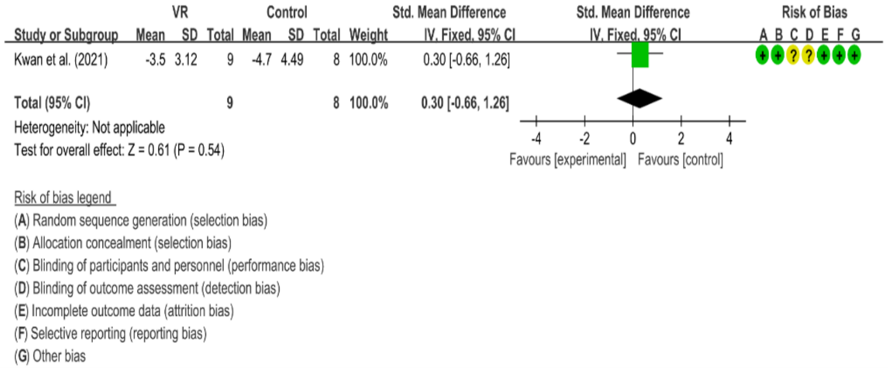

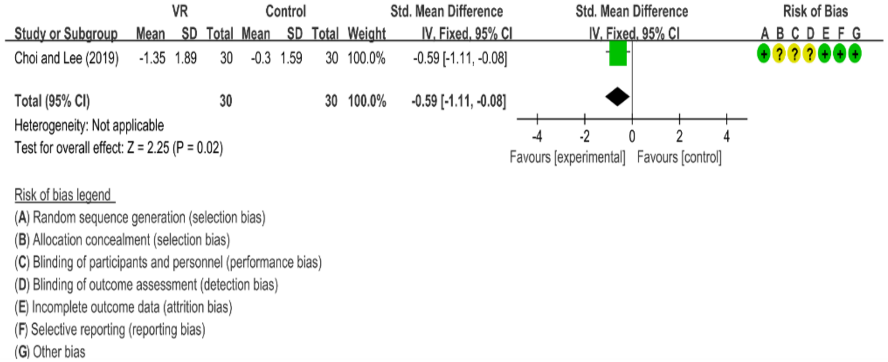

(2)A vs D

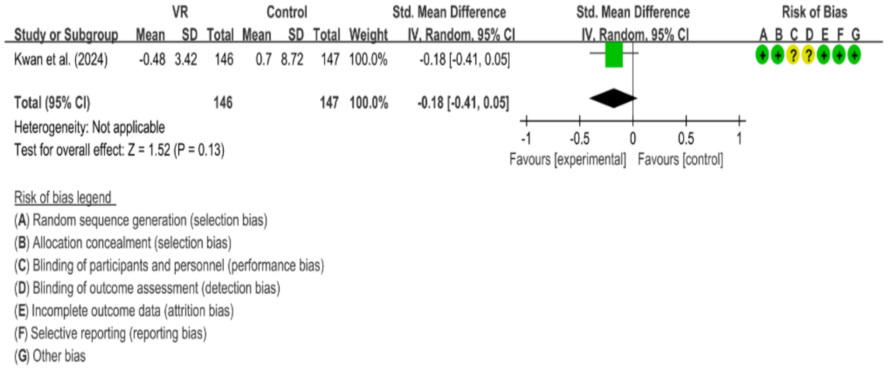

(4)B vs D

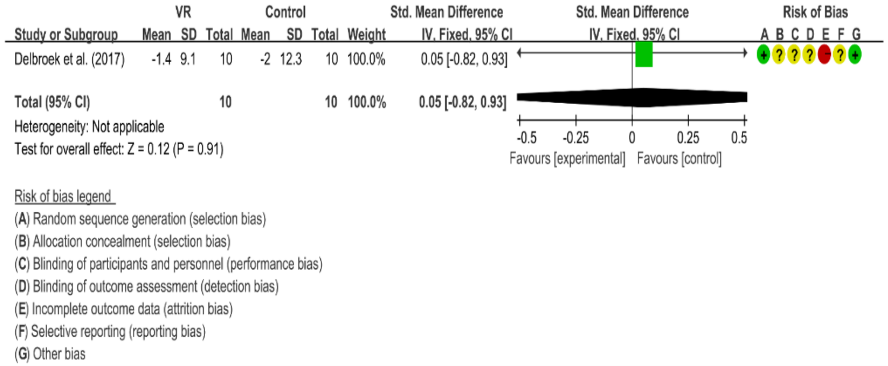

Figure S15. Forest plots of TUG outcomes across intervention comparisons. (1) Comparison between Fully Immersive VR (A) and Active Control (C); (2) Comparison between Fully Immersive VR (A) and Passive Control (D); (3) Comparison between Partially Immersive VR (B) and Active Control (C); (4) Comparison between Partially Immersive VR (B) and Passive Control (D); Effect sizes are presented as standardized mean differences (SMDs) with 95% confidence intervals (CIs). A positive SMD indicates greater improvement in the VR or active intervention group compared to the control. The included studies are: (Kwan et al. (2021), Choi and Lee (2019), Kwan et al. (2024), Delbroek et al. (2017)).

TUG

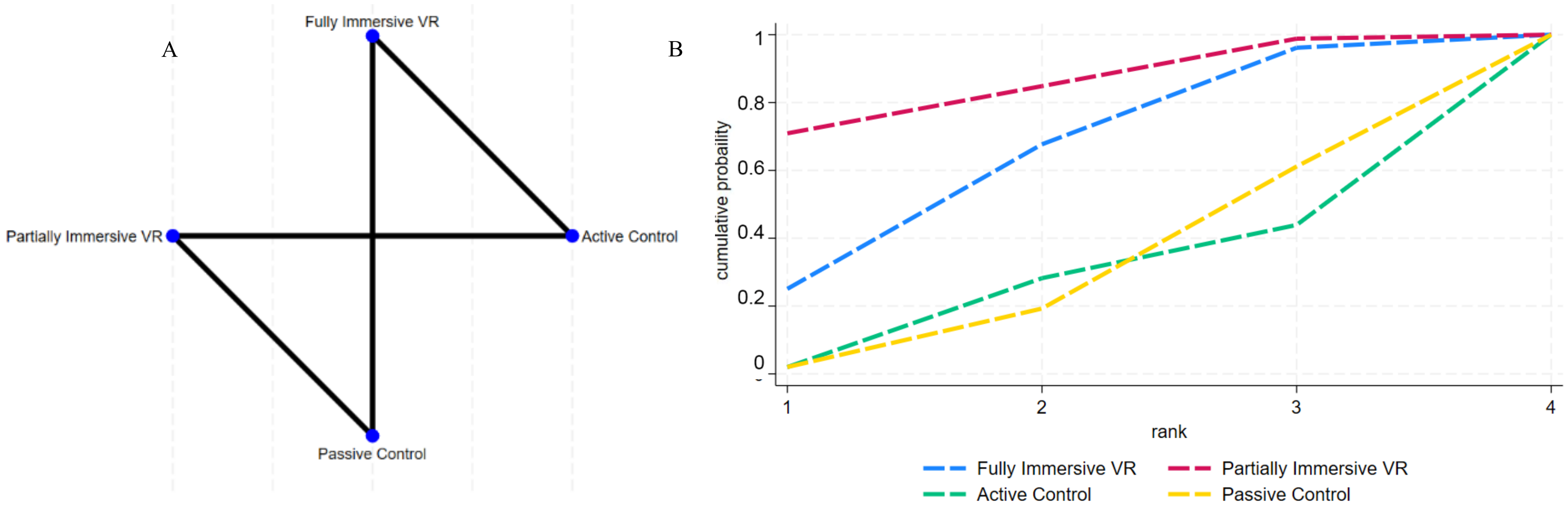

C

**Partially Immersive VR**			
**0.04 (−0.56,0.63)**	**Fully Immersive VR**		
**0.12 (−0.55,0.80)**	**0.08 (−0.36,0.53)**	**Passive Control**	
**0.42 (−0.22,1.05)**	**0.38 (−0.35,1.11)**	**0.29 (−0.46,1.05)**	**Active Control**

Figure S16. Network meta-analysis results of TUG outcomes. (A) Network plot showing direct and indirect comparisons; node size reflects sample size, and edge thickness represents the number of comparisons. (B) SUCRA ranking plot of each intervention, where Surface Under the Cumulative Ranking Curve indicate better relative efficacy. (C) League table presenting the pairwise standardized mean differences (SMDs) and 95% confidence intervals (CIs) for all comparisons among interventions.

Grip Strength

(1)A vs D

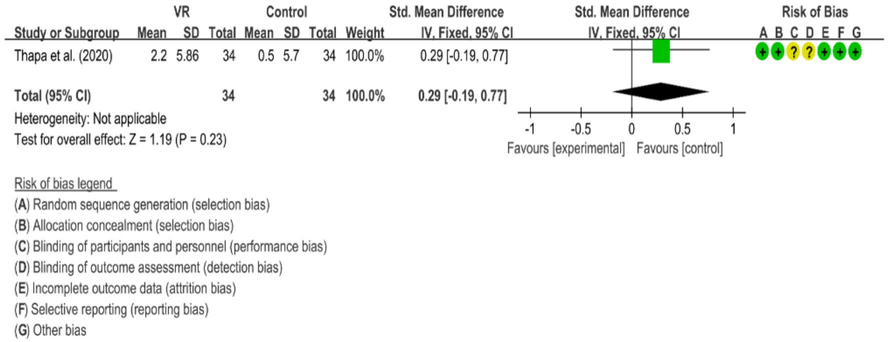

(2)B vs D

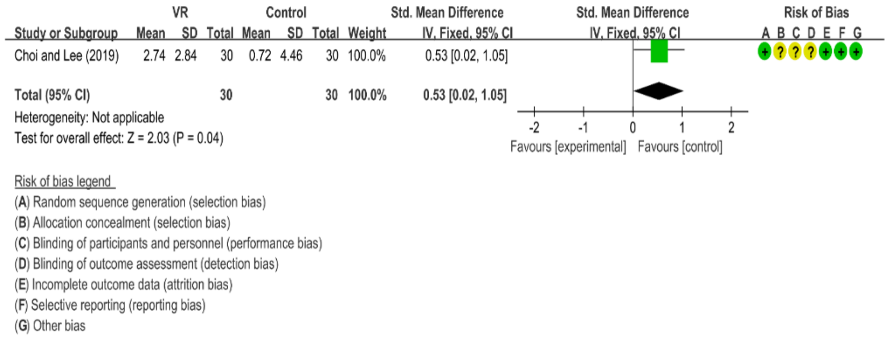

Figure S17. Forest plots of Grip Strength outcomes across intervention comparisons. (1) Comparison between Fully Immersive VR (A) and Active Control (C); (2) Comparison between Fully Immersive VR (A) and Passive Control (D); (3) Comparison between Partially Immersive VR (B) and Active Control (C);(4) Comparison between Partially Immersive VR (B) and Passive Control (D); Effect sizes are presented as standardized mean differences (SMDs) with 95% confidence intervals (CIs). A positive SMD indicates greater improvement in the VR or active intervention group compared to the control. The included studies are: (Thapa et al. (2020), Choi and Lee (2019)).

FFP

(1)A vs C

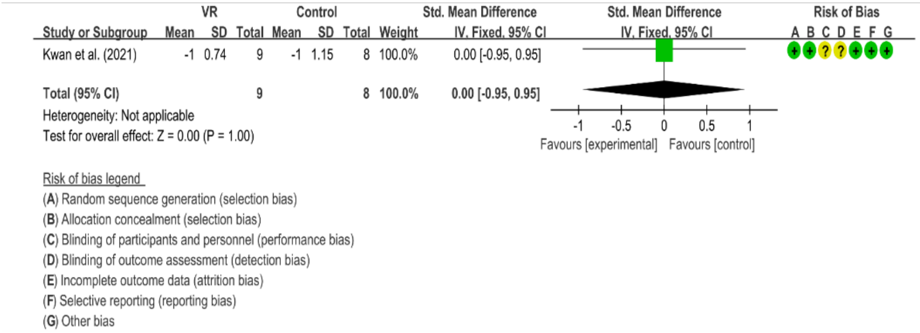

(2)A vs D

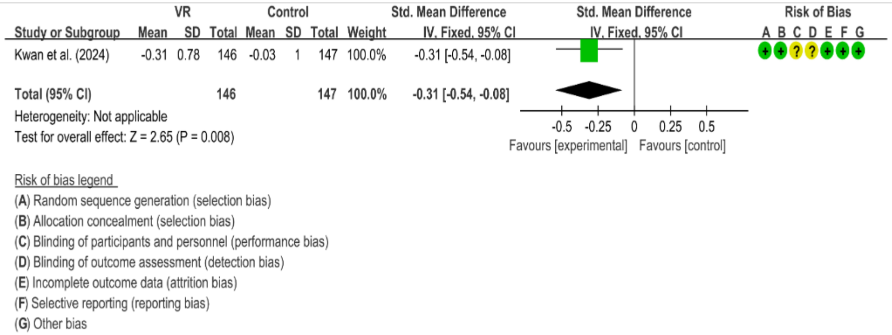

Figure S18. Forest plots of FFP outcomes across intervention comparisons. (1) Comparison between Fully Immersive VR (A) and Active Control (C); (2) Comparison between Fully Immersive VR (A) and Passive Control (D); Effect sizes are presented as standardized mean differences (SMDs) with 95% confidence intervals (CIs). A positive SMD indicates greater improvement in the VR or active intervention group compared to the control. The included studies are: (Kwan et al. (2021), Kwan et al. (2024)).

IADL

(1)A vs C

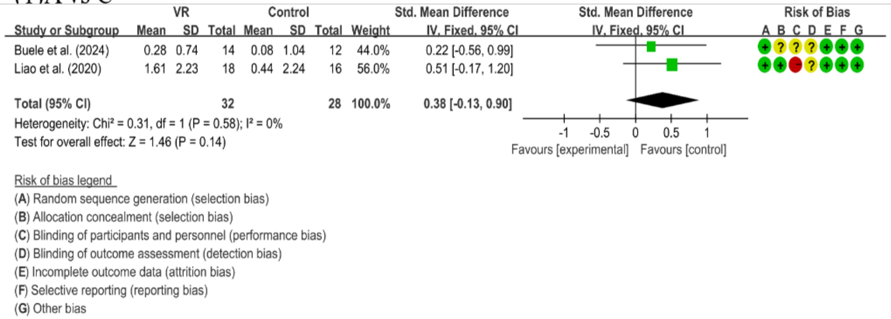

(2)B vs D

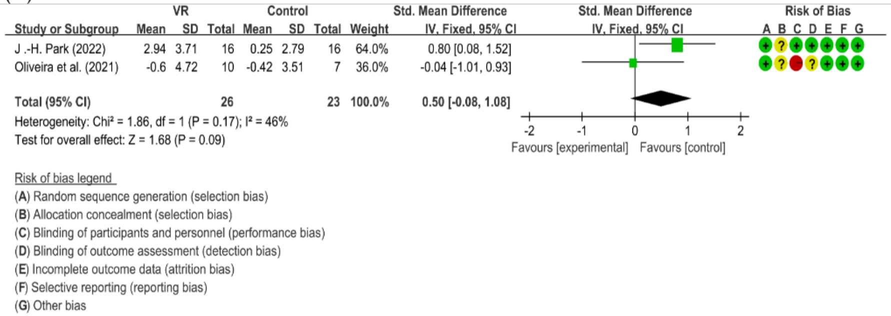

Figure S19. Forest plots of IADL outcomes across intervention comparisons. (1) Comparison between Fully Immersive VR (A) and Active Control (C); (2) Comparison between Partially Immersive VR (B) and Passive Control (D); Effect sizes are presented as standardized mean differences (SMDs) with 95% confidence intervals (CIs). A positive SMD indicates greater improvement in the VR or active intervention group compared to the control. The included studies are: (Buele et al. (2024), Liao et al. (2020), J.-H. Park (2022), Oliveira et al. (2021)).

SGDS

(1)A vs C

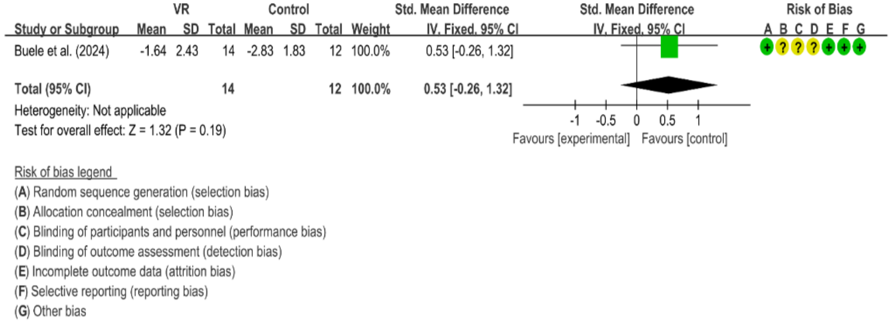

(2)A vs D

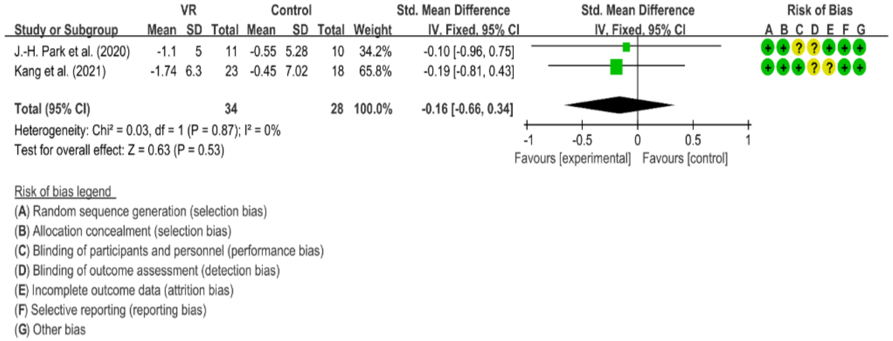

Figure S20. Forest plots of SGDS outcomes across intervention comparisons. (1) Comparison between Fully Immersive VR (A) and Active Control (C); (2) Comparison between Fully Immersive VR (A) and Passive Control (D); Effect sizes are presented as standardized mean differences (SMDs) with 95% confidence intervals (CIs). A positive SMD indicates greater improvement in the VR or active intervention group compared to the control. The included studies are: (Buele et al. (2024), J.-H. Park et al. (2020), Kang et al. (2021)).
