# Supplementary material for: Virtual Reality-Based Cognitive and Physical Interventions in Cognitive Impairment: A Network Meta-Analysis of Immersion Level Effects
Source: Behav Sci (Basel). 2025 Nov 22;15(12):1610. doi: 10.3390/bs15121610 (PMC12730044; doi:10.3390/bs15121610)
Supplement: Supplementary file 1 [file behavsci-15-01610-s001.zip › Supplementary table.pdf]

Table S1. Database Yield and Screening Steps Summary
(Complementary to PRISMA Flow Diagram)

Steps	Specific Description	Search Query / Description	Records Retrieved
**1.Identification (Database Search)**	PubMed	(((("Cognitive Dysfunction"[Mesh]) OR (((((((((((((((((((((((((Cognitive Dysfunctions[Title/Abstract]) OR (Dysfunction, Cognitive[Title/Abstract])) OR (Dysfunctions, Cognitive[Title/Abstract])) OR (Cognitive Disorder[Title/Abstract])) OR (Cognitive Disorders[Title/Abstract])) OR (Disorder, Cognitive[Title/Abstract])) OR (Disorders, Cognitive[Title/Abstract])) OR (Cognitive Impairments[Title/Abstract])) OR (Cognitive Impairment[Title/Abstract])) OR (Impairment, Cognitive[Title/Abstract])) OR (Impairments, Cognitive[Title/Abstract])) OR (Mild Cognitive Impairment[Title/Abstract])) OR (Cognitive Impairment, Mild[Title/Abstract])) OR (Cognitive Impairments, Mild[Title/Abstract])) OR (Impairment, Mild Cognitive[Title/Abstract])) OR (Impairments, Mild Cognitive[Title/Abstract])) OR (Mild Cognitive Impairments[Title/Abstract])) OR (Cognitive Decline[Title/Abstract])) OR (Cognitive Declines[Title/Abstract])) OR (Decline, Cognitive[Title/Abstract])) OR (Declines, Cognitive[Title/Abstract])) OR (Mental Deterioration[Title/Abstract])) OR (Deterioration, Mental[Title/Abstract])) OR (Deteriorations, Mental[Title/Abstract])) OR (Mental Deteriorations[Title/Abstract]))) OR (("Alzheimer Disease"[Mesh]) OR (((((((((((((((((((((((((((((((((((Alzheimer Syndrome[Title/Abstract]) OR (Alzheimer-Type Dementia (ATD[Title/Abstract]))) OR (Alzheimer Type Dementia (ATD[Title/Abstract]))) OR (Dementia, Alzheimer-Type (ATD[Title/Abstract]))) OR (Alzheimer's Diseases[Title/Abstract])) OR (Alzheimer Diseases[Title/Abstract])) OR (Alzheimers Diseases[Title/Abstract])) OR (Alzheimer Dementia[Title/Abstract])) OR (Alzheimer Dementias[Title/Abstract])) OR (Dementia, Alzheimer[Title/Abstract])) OR (Alzheimer's Disease[Title/Abstract])) OR (Dementia, Senile[Title/Abstract]))	140

OR (Senile Dementia[Title/Abstract])) OR (Dementia, Alzheimer Type[Title/Abstract])) OR (Alzheimer Type Dementia[Title/Abstract])) OR (Senile Dementia, Alzheimer Type[Title/Abstract])) OR (Alzheimer Type Senile Dementia[Title/Abstract])) OR (Primary Senile Degenerative Dementia[Title/Abstract])) OR (Alzheimer Sclerosis[Title/Abstract])) OR (Sclerosis, Alzheimer[Title/Abstract])) OR (Dementia, Primary Senile Degenerative[Title/Abstract])) OR (Dementia, Presenile[Title/Abstract])) OR (Presenile Dementia[Title/Abstract])) OR (Acute Confusional Senile Dementia[Title/Abstract])) OR (Senile Dementia, Acute Confusional[Title/Abstract])) OR (Alzheimer Disease, Early Onset[Title/Abstract])) OR (Early Onset Alzheimer Disease[Title/Abstract])) OR (Presenile Alzheimer Dementia[Title/Abstract])) OR (Alzheimer Disease, Late Onset[Title/Abstract])) OR (Late Onset Alzheimer Disease[Title/Abstract])) OR (Alzheimer's Disease, Focal Onset[Title/Abstract])) OR (Focal Onset Alzheimer's Disease[Title/Abstract])) OR (Familial Alzheimer Disease (FAD[Title/Abstract]))) OR (Alzheimer Disease, Familial (FAD[Title/Abstract]))) OR (Familial Alzheimer Diseases (FAD[Title/Abstract]))))) AND ((("Virtual Reality"[Mesh]) OR ((((((((((((Reality, Virtual[Title/Abstract]) OR (Virtual Reality, Educational[Title/Abstract])) OR (Educational Virtual Realities[Title/Abstract])) OR (Educational Virtual Reality[Title/Abstract])) OR (Reality, Educational Virtual[Title/Abstract])) OR (Virtual Realities, Educational[Title/Abstract])) OR (Virtual Reality, Instructional[Title/Abstract])) OR (Instructional Virtual Realities[Title/Abstract])) OR (Instructional Virtual Reality[Title/Abstract])) OR (Realities, Instructional Virtual[Title/Abstract])) OR (Reality, Instructional Virtual[Title/Abstract])) OR (Virtual Realities, Instructional[Title/Abstract]))) OR (("Virtual Reality Exposure Therapy"[Mesh]) OR ((((((((((((((((((((Virtual Reality Immersion Therapy[Title/Abstract]) OR (Virtual Reality Therapy[Title/Abstract])) OR (Reality Therapies, Virtual[Title/Abstract])) OR (Reality Therapy, Virtual[Title/Abstract])) OR (Therapies, Virtual Reality[Title/Abstract])) OR (Therapy, Virtual

	Reality[Title/Abstract])) OR (Virtual Reality Therapies[Title/Abstract])) OR (Exergaming[Title/Abstract])) OR (Exergaming [Title/Abstract])) OR (Virtual Reality Exercise[Title/Abstract])) OR (Exercises, Virtual Reality[Title/Abstract])) OR (Exercise, Virtual Reality[Title/Abstract])) OR (Virtual Reality Exercises[Title/Abstract])) OR (Active-Video Gaming[Title/Abstract])) OR (Active Video Gaming[Title/Abstract])) OR (Active-Video Gamings[Title/Abstract])) OR (Gaming, Active-Video[Title/Abstract])) OR (Gamings, Active-Video[Title/Abstract])) OR (Exergames[Title/Abstract])) OR (Exergame[Title/Abstract]))))) AND (Randomized Controlled Trial [Publication Type] OR randomized [Title/Abstract] OR placebo [Title/Abstract])	
Cochrane library	(Cognitive Dysfunctions):ab,ti,kw OR (Dysfunction, Cognitive):ab,ti,kw OR (Dysfunctions, Cognitive):ab,ti,kw OR (Cognitive Disorder):ab,ti,kw OR (Cognitive Disorders):ab,ti,kw OR (Disorder, Cognitive):ab,ti,kw OR (Disorders, Cognitive):ab,ti,kw OR (Cognitive Impairments):ab,ti,kw OR (Cognitive Impairment):ab,ti,kw OR (Impairment, Cognitive):ab,ti,kw OR (Impairments, Cognitive):ab,ti,kw OR (Mild Cognitive Impairment):ab,ti,kw OR (Cognitive Impairment, Mild):ab,ti,kw OR (Cognitive Impairments, Mild):ab,ti,kw OR (Impairment, Mild Cognitive):ab,ti,kw OR (Impairments, Mild Cognitive):ab,ti,kw OR (Mild Cognitive Impairments):ab,ti,kw OR (Cognitive Decline):ab,ti,kw OR (Cognitive Declines):ab,ti,kw OR (Decline, Cognitive):ab,ti,kw OR (Declines, Cognitive):ab,ti,kw OR (Mental Deterioration):ab,ti,kw OR (Deterioration, Mental):ab,ti,kw OR (Deteriorations, Mental):ab,ti,kw OR (Mental Deteriorations):ab,ti,kw OR (Alzheimer Syndrome):ab,ti,kw OR (Alzheimer-Type Dementia (ATD)):ab,ti,kw OR (Alzheimer Type Dementia (ATD)):ab,ti,kw OR (Dementia, Alzheimer-Type (ATD)):ab,ti,kw OR (Alzheimer's Diseases):ab,ti,kw OR (Alzheimer Diseases):ab,ti,kw OR (Alzheimers Diseases):ab,ti,kw OR (Alzheimer Dementia):ab,ti,kw OR (Alzheimer Dementias):ab,ti,kw OR (Dementia, Alzheimer):ab,ti,kw OR (Alzheimer's	707

	Disease):ab,ti,kw OR (Dementia, Senile):ab,ti,kw OR (Senile Dementia):ab,ti,kw OR (Dementia, Alzheimer Type):ab,ti,kw OR (Alzheimer Type Dementia):ab,ti,kw OR (Senile Dementia, Alzheimer Type):ab,ti,kw OR (Alzheimer Type Senile Dementia):ab,ti,kw OR (Primary Senile Degenerative Dementia):ab,ti,kw OR (Alzheimer Sclerosis):ab,ti,kw OR (Sclerosis, Alzheimer):ab,ti,kw OR (Dementia, Primary Senile Degenerative):ab,ti,kw OR (Dementia, Presenile):ab,ti,kw OR (Presenile Dementia):ab,ti,kw OR (Acute Confusional Senile Dementia):ab,ti,kw OR (Senile Dementia, Acute Confusional):ab,ti,kw OR (Alzheimer Disease, Early Onset):ab,ti,kw OR (Early Onset Alzheimer Disease):ab,ti,kw OR (Presenile Alzheimer Dementia):ab,ti,kw OR (Alzheimer Disease, Late Onset):ab,ti,kw OR (Late Onset Alzheimer Disease):ab,ti,kw OR (Alzheimer's Disease, Focal Onset):ab,ti,kw OR (Focal Onset Alzheimer's Disease):ab,ti,kw OR (Familial Alzheimer Disease (FAD)):ab,ti,kw OR (Alzheimer Disease, Familial (FAD)):ab,ti,kw OR (Familial Alzheimer Diseases (FAD)):ab,ti,kw AND (Reality, Virtual):ab,ti,kw OR (Virtual Reality, Educational):ab,ti,kw OR (Educational Virtual Realities):ab,ti,kw OR (Educational Virtual Reality):ab,ti,kw OR (Reality, Educational Virtual):ab,ti,kw OR (Virtual Realities, Educational):ab,ti,kw OR (Virtual Reality, Instructional):ab,ti,kw OR (Instructional Virtual Realities):ab,ti,kw OR (Instructional Virtual Reality):ab,ti,kw OR (Realities, Instructional Virtual):ab,ti,kw OR (Reality, Instructional Virtual):ab,ti,kw OR (Virtual Realities, Instructional):ab,ti,kw OR (Virtual Reality Exposure Therapy):ab,ti,kw OR (Virtual Reality Immersion Therapy):ab,ti,kw OR (Virtual Reality Therapy):ab,ti,kw OR (Reality Therapies, Virtual):ab,ti,kw OR (Reality Therapy, Virtual):ab,ti,kw OR (Therapies, Virtual Reality):ab,ti,kw OR (Therapy, Virtual Reality):ab,ti,kw OR (Virtual Reality Therapies):ab,ti,kw OR (Exergaming):ab,ti,kw OR (Exergamings):ab,ti,kw OR (Virtual Reality Exercise):ab,ti,kw OR (Exercises, Virtual Reality):ab,ti,kw OR (Exercise, Virtual Reality):ab,ti,kw OR (Virtual Reality Exercises):ab,ti,kw OR (Active-Video Gaming):ab,ti,kw OR (Active Video Gaming):ab,ti,kw OR

	(Active-Video Gamings):ab,ti,kw OR (Gaming, Active-Video):ab,ti,kw OR (Gamings, Active-Video):ab,ti,kw OR (Exergames):ab,ti,kw OR (Exergame):ab,ti,kw AND (Randomized Controlled Trial):ab,ti,kw OR (randomized):ab,ti,kw OR (placebo):ab,ti,kw OR (RCT):ab,ti,kw	
Embase	'Cognitive Dysfunctions':ab,ti OR 'Dysfunction, Cognitive':ab,ti OR 'Dysfunctions, Cognitive':ab,ti OR 'Cognitive Disorder':ab,ti OR 'Cognitive Disorders':ab,ti OR 'Disorder, Cognitive':ab,ti OR 'Disorders, Cognitive':ab,ti OR 'Cognitive Impairments':ab,ti OR 'Cognitive Impairment':ab,ti OR 'Impairment, Cognitive':ab,ti OR 'Impairments, Cognitive':ab,ti OR 'Mild Cognitive Impairment':ab,ti OR 'Cognitive Impairment, Mild':ab,ti OR 'Cognitive Impairments, Mild':ab,ti OR 'Impairment, Mild Cognitive':ab,ti OR 'Impairments, Mild Cognitive':ab,ti OR 'Mild Cognitive Impairments':ab,ti OR 'Cognitive Decline':ab,ti OR 'Cognitive Declines':ab,ti OR 'Decline, Cognitive':ab,ti OR 'Declines, Cognitive':ab,ti OR 'Mental Deterioration':ab,ti OR 'Deterioration, Mental':ab,ti OR 'Deteriorations, Mental':ab,ti OR 'Mental Deteriorations':ab,ti OR 'Alzheimer Syndrome':ab,ti OR 'Alzheimer-Type Dementia (ATD)':ab,ti OR 'Alzheimer Type Dementia (ATD)':ab,ti OR 'Dementia, Alzheimer-Type (ATD)':ab,ti OR 'Alzheimer Diseases':ab,ti OR 'Alzheimer Diseases':ab,ti OR 'Alzheimers Diseases':ab,ti OR 'Alzheimer Dementia':ab,ti OR 'Alzheimer Dementias':ab,ti OR 'Dementia, Alzheimer':ab,ti OR 'Alzheimer Disease':ab,ti OR 'Dementia, Senile':ab,ti OR 'Senile Dementia':ab,ti OR 'Dementia, Alzheimer Type':ab,ti OR 'Alzheimer Type Dementia':ab,ti OR 'Senile Dementia, Alzheimer Type':ab,ti OR 'Alzheimer Type Senile Dementia':ab,ti OR 'Primary Senile Degenerative Dementia':ab,ti OR 'Alzheimer Sclerosis':ab,ti OR 'Sclerosis, Alzheimer':ab,ti OR 'Dementia, Primary Senile Degenerative':ab,ti OR 'Dementia, Presenile':ab,ti OR 'Presenile Dementia':ab,ti OR 'Acute Confusional Senile Dementia':ab,ti OR 'Senile Dementia, Acute Confusional':ab,ti OR 'Alzheimer Disease, Early Onset':ab,ti OR 'Early Onset Alzheimer Disease':ab,ti OR 'Presenile Alzheimer Dementia':ab,ti	251

	OR 'Alzheimer Disease, Late Onset':ab,ti OR 'Late Onset Alzheimer Disease':ab,ti OR 'Alzheimer Disease, Focal Onset':ab,ti OR 'Focal Onset Alzheimer Disease':ab,ti OR 'Familial Alzheimer Disease (FAD)':ab,ti OR 'Alzheimer Disease, Familial (FAD)':ab,ti OR 'Familial Alzheimer Diseases (FAD)':ab,ti AND 'Reality, Virtual':ab,ti OR 'Virtual Reality, Educational':ab,ti OR 'Educational Virtual Realities':ab,ti OR 'Educational Virtual Reality':ab,ti OR 'Reality, Educational Virtual':ab,ti OR 'Virtual Realities, Educational':ab,ti OR 'Virtual Reality, Instructional':ab,ti OR 'Instructional Virtual Realities':ab,ti OR 'Instructional Virtual Reality':ab,ti OR 'Realities, Instructional Virtual':ab,ti OR 'Reality, Instructional Virtual':ab,ti OR 'Virtual Realities, Instructional':ab,ti OR 'Virtual Reality Exposure Therapy':ab,ti OR 'Virtual Reality Immersion Therapy':ab,ti OR 'Virtual Reality Therapy':ab,ti OR 'Reality Therapies, Virtual':ab,ti OR 'Reality Therapy, Virtual':ab,ti OR 'Therapies, Virtual Reality':ab,ti OR 'Therapy, Virtual Reality':ab,ti OR 'Virtual Reality Therapies':ab,ti OR 'Exergaming':ab,ti OR 'Exergamings':ab,ti OR 'Virtual Reality Exercise':ab,ti OR 'Exercises, Virtual Reality':ab,ti OR 'Exercise, Virtual Reality':ab,ti OR 'Virtual Reality Exercises':ab,ti OR 'Active-Video Gaming':ab,ti OR 'Active Video Gaming':ab,ti OR 'Active-Video Gamings':ab,ti OR 'Gaming, Active-Video':ab,ti OR 'Gamings, Active-Video':ab,ti OR 'Exergames':ab,ti OR 'Exergame':ab,ti AND 'Randomized Controlled Trial':ab,ti OR 'Randomized':ab,ti OR 'Placebo':ab,ti OR 'RCT':ab,ti	
Web of science	Cognitive Dysfunction OR Cognitive Dysfunctions OR Dysfunction, Cognitive OR Dysfunctions, Cognitive OR Cognitive Disorder OR Cognitive Disorders OR Disorder, Cognitive OR Disorders, Cognitive OR Cognitive Impairments OR Cognitive Impairment OR Impairment, Cognitive OR Impairments, Cognitive OR Mild Cognitive Impairment OR Cognitive Impairment, Mild OR Cognitive Impairments, Mild OR Impairment, Mild Cognitive OR Impairments, Mild Cognitive OR Mild Cognitive Impairments OR Cognitive Decline OR Cognitive Declines OR Decline, Cognitive OR Declines, Cognitive OR Mental Deterioration OR	2101

	Deterioration, Mental OR Deteriorations, Mental OR Mental Deteriorations OR Alzheimer Disease OR Alzheimer Syndrome OR Alzheimer-Type Dementia (ATD) OR Alzheimer Type Dementia (ATD) OR Dementia, Alzheimer-Type (ATD) OR Alzheimer's Diseases OR Alzheimer Diseases OR Alzheimers Diseases OR Alzheimer Dementia OR Alzheimer Dementias OR Dementia, Alzheimer OR Alzheimer's Disease OR Dementia, Senile OR Senile Dementia OR Dementia, Alzheimer Type OR Alzheimer Type Dementia OR Senile Dementia, Alzheimer Type OR Alzheimer Type Senile Dementia OR Primary Senile Degenerative Dementia OR Alzheimer Sclerosis OR Sclerosis, Alzheimer OR Dementia, Primary Senile Degenerative OR Dementia, Presenile OR Presenile Dementia OR Acute Confusional Senile Dementia OR Senile Dementia, Acute Confusional OR Alzheimer Disease, Early Onset OR Early Onset Alzheimer Disease OR Presenile Alzheimer Dementia OR Alzheimer Disease, Late Onset OR Late Onset Alzheimer Disease OR Alzheimer's Disease, Focal Onset OR Focal Onset Alzheimer's Disease OR Familial Alzheimer Disease (FAD) OR Alzheimer Disease, Familial (FAD) OR Familial Alzheimer Diseases (FAD) AND Virtual reality OR Reality, Virtual OR Virtual Reality, Educational OR Educational Virtual Realities OR Educational Virtual Reality OR Reality, Educational Virtual OR Virtual Realities, Educational OR Virtual Reality, Instructional OR Instructional Virtual Realities OR Instructional Virtual Reality OR Realities, Instructional Virtual OR Reality, Instructional Virtual OR Virtual Realities, Instructional OR Virtual Reality Exposure Therapy OR Cognitive Impairments, Mild OR Virtual Reality Immersion Therapy OR Virtual Reality Therapy OR Reality Therapies, Virtual OR Reality Therapy, Virtual OR Therapies, Virtual Reality OR Therapy, Virtual Reality OR Virtual Reality Therapies OR Exergaming OR Exergamings OR Virtual Reality Exercise OR Exercises, Virtual Reality OR Exercise, Virtual Reality OR Virtual Reality Exercises OR Active-Video Gaming OR Active Video Gaming OR Active-Video Gamings OR Gaming, Active-Video OR Gamings, Active-Video OR Exergames

		OR Exergame AND Randomized Controlled Trial OR Randomized OR Placebo OR RCT	
	**Total Retrieved**	-	3199
**2.Remove Duplicates**	After merging all database results	Removed via EndNote X9 duplicate removal tool	2695
**3.Initial Screening (Title/Abstract)**	Excluded: Meta-analyses, systematic reviews, animal experiments	Exclusion reason: Study type not eligible	417
	**Remaining after initial screening**	-	2278
	Excluded: Inconsistent research content	Exclusion reason: Topic irrelevant to the research question	2210
**4.Full-Text Eligibility Assessment**	Need full-text retrieval	-	68
	Excluded: Inconsistent intervention measures	Intervention not matching (e.g., non-VR control, wrong VR type)	12

	Excluded: Inconsistent experimental design	Study design (e.g., cross-sectional, non-randomized) not meeting inclusion criteria	12
	Excluded: Inconsistent outcome variables	Outcomes not including [key outcome measures]	10
	Excluded: Inconsistent target population	Population not [target population, e.g., patients with specific condition]	14
	**Total excluded at full-text stage**	-	48
**5.Final Inclusion**	Included in Meta-analysis	All met the study's inclusion/exclusion criteria and PRISMA-NMA guidelines	20

Footnotes:

1. *This table details the quantitative data and exclusion reasons for each screening stage, complementing the PRISMA flow diagram to enhance research reproducibility.*
2. *Search strategies for each database are consistent and available upon request from the corresponding author.*

Table S2. VR Intervention Protocols and Outcome Measures

Study (Year)	VR Immersion Level	Experimental Group Protocol	Control Type	Control Group Protocol	Follow-up	Primary Outcome Measures
Li et al. (2025)	Partially immersive	Device: Nintendo Switch<br>Tasks: Motion-sensing exergames (WarioWare: Move It!)	Active control	Physical exercise + Horticulture	6 months	CASI, MMSE, MoCA
Kwan et al. (2024)	Fully immersive	Device: HTC Vive Focus Plus HMD<br>Tasks: 8 functional ADL simulations (bus stop navigation, grocery shopping)<br>Dose: 30-min VR/session	Passive control	Usual care	None	MoCA, FFP, TUG, SCWT, DST, TMT
Buele et al. (2024)	Fully immersive	Device: Oculus Quest 2<br>Tasks: Virtual kitchen ingredient search with difficulty progression	Active control	Card-based cognitive training	None	MoCA, IADL, SGDS
Chiu et al. (2023)	Fully immersive	Device: HMD system<br>Tasks: Underwater cognitive tasks (color/shape identification, calculation)	Passive control	Routine care	None	CASI, MMSE
Arshad et al. (2023)	Partially immersive	Device: Xbox 360 Kinect<br>Tasks: Dr. Kawashima cognitive games (logic/memory/math)	Active control	Stretching+ Resistance training	None	MMSE, MoCA, TMT, VFT
J. G. Yang et al. (2022)	Fully immersive	Device: Oculus Quest<br>Tasks: Multi-domain cognitive training (attention/working memory/executive function) | Aerobic + Resistance Training	Passive control	Health education lectures	None	MMSE, TMT, Grip strength
J.-H. Park (2022)	Partially immersive	Device: Tablet-based virtual supermarket application	Passive control	No intervention	None	IADL
Oliveira et	Partially	Device: Computer + SLB software	Passive control	Standard institutional care	None	MMSE, IADL,

al (2021)	immersive	Tasks: Simulated IADL tasks (virtual kitchen, pharmacy)				FAB
Kwan et al. (2021)	Fully immersive	Device: HTC Vive<br>Tasks:Dual-task cycling (calculation/memory) + functional activities	Active control	Tablet games + Cycling	None	MoCA, FFP, TUG
Kang et al. (2021)	Fully immersive	Device: Oculus Rift CV1<br>Tasks: Multi-domain cognitive training (attention/executive function/visuospatial)	Passive control	Conventional pharmacotherapy	None	GDS
Thapa et al. (2020)	Fully immersive	Device: Oculus Quest<br>Tasks: Juice preparation, bird shooting, house memory recall	Passive control	Health education lectures	None	MMSE, TMT, SDST, Grip strength
J.S. Park et al. (2020)	Partially immersive	Device: MOTOCOG® system<br>Tasks: Simulated daily activities (driving, shopping, cooking)	Active control	Tabletop activities (puzzles, blocks)	None	MoCA, TMT, DST
J.-H. Park et al. (2020)	Fully immersive	Device: HTC Vive<br>Tasks: Attention training (crow shooting), executive function (shopping), memory (fireworks)	Passive control	Maintain daily activities	None	MMSE, VFT, DST, SGDS
Liao et al. (2020)	Fully immersive	Device: Kinect + Vive HMD<br>Tasks: Physical exercise (Tai Chi) + VR-IADL tasks (cooking, shopping)	Active control	Resistance + Aerobic + Dual-task training	None	MoCA, IADL
H.-L. Yang et al. (2019)	Partially immersive	Device: Computer + CogniPlus software<br>Tasks: Visual/spatial memory training	Active control	E-books + Puzzle games	3 months	MMSE, MoCA, DST-Backward
Liao et al. (2019)	Fully immersive	Device: VR HMD<br>Tasks: Physical exercise + VR-IADL tasks (metro ticketing, cooking)	Active control	Resistance/Aerobic/Balance + Dual-task	None	SCWT, TMT, Gait speed
Choi and Lee (2019)	Partially immersive	Device: 100-inch screen + paddle<br>Tasks: Virtual kayaking with directional	Active control	Home-based physical exercise	None	MoCA, TUG, Grip strength

		challenges					
Delbroek et al. (2017)	Partially immersive	Device: Bio Rescue platform Tasks: Balance training + cognitive tasks (memory cards)		Passive control	Routine care	None	MoCA, TUG
Serino et al. (2017)	Partially immersive	Device: NeuroVirtual 3D + joystick Tasks: Object location memory in virtual city		Active control	Traditional cognitive stimulation	None	VFT, FAB, DST
Baldimtsi et al. (2023)	Fully immersive	Device: Oculus Go HMD Tasks: Dual-task cycling (calculation/memory games)	Non-VR Cycling with Computational Tasks	Passive control	No intervention	None	MMSE, TMT-B, DST-Forward

Abbreviations: ADL = Activities of Daily Living; MoCA = Montreal Cognitive Assessment; FFP = Fried Frailty Phenotype; TUG = Timed Up and Go test; SCWT = Stroop Color and Word Test; DST = Digit Span Test; TMT = Trail Making Test; IADL = Instrumental Activities of Daily Living; SGDS = Short Geriatric Depression Scale; VFT = Verbal Fluency Test; FAB = Frontal Assessment Battery; SDST = Symbol Digit Substitution Test; CASI = Cognitive Abilities Screening Instrument; MMSE = Mini-Mental State Examination; GDS = Geriatric Depression Scale

References

(Arshad et al., 2023) Arshad, H., Khattak, H. G., & Anwar, K. (2023). Effect of exergames by using Xbox 360 Kinect on cognition of older adults with mild cognitive impairment. Rawal Medical Journal, 48(4), 994–998. http://doi.org/10.5455/rmj.20210821031024.

(Baldimtsi et al., 2023) Baldimtsi, E., Mouzakidis, C., Karathanasi, E. M., Verykouki, E., Hassandra, M., Galanis, E., Hatzigeorgiadis, A., Goudas, M., Zikas, P., Evangelou, G., Papagiannakis, G., Bellis, G., Kokkotis, C., Tsatalas, T., Giakas, G., Theodorakis, Y., & Tsolaki, M. (2023). Effects of virtual reality physical and cognitive training intervention on cognitive abilities of elders with mild cognitive impairment. Journal of Alzheimer's Disease Reports, 7(1), 1475–1490. http://doi.org/10.3233/ADR-230099. PMCID: PMC10789285.

(Buele et al., 2024) Buele, J., Avilés-Castillo, F., Valle Soto, C. D., Varela-Aldás, J., & Palacios-Navarro, G. (2024). Effects of a dual intervention (motor and virtual reality-based cognitive) on cognition in patients with mild cognitive impairment: A single-blind, randomized controlled trial. Journal of Neuroengineering and Rehabilitation, 21(1), 130. http://doi.org/10.1186/s12984-024-01422-w.

(Chiu et al., 2023) Chiu, H. M., Hsu, M. C., & Ouyang, W. C. (2023). Effects of incorporating virtual reality training intervention into health care on cognitive function and wellbeing in older adults with cognitive impairment: A randomized controlled trial. International Journal of Human-Computer Studies, 170, 1–12. http://doi.org/10.1016/j.ijhcs.2022.102957.

(Choi & Lee, 2019) Choi, W., & Lee, S. (2019). Virtual kayak paddling exercise improves postural balance, muscle performance, and cognitive function in older adults with mild cognitive impairment: A randomized controlled trial. Journal of Aging and Physical Activity, 27, 861–870. https://doi.org/10.1123/jap.2018-0020.

(Delbroek et al., 2017) Delbroek, T., Vermeylen, W., & Spildooren, J. (2017). The effect of cognitive-motor dual task training with the biorescue force platform on cognition, balance and dual task performance in institutionalized older adults: A randomized controlled trial. Journal of Physical Therapy Science, 29(7), 1137–1143. http://doi.org/10.1589/jpts.29.1137. PMCID: PMC5509577.

(Kang et al., 2021) Kang, J. M., Kim, N., Lee, S. Y., Woo, S. K., Park, G., Yeon, B. K., Park, J. W., Youn, J., Ryu, S., Lee, J., & Cho, S.-J. (2021a). Effect of cognitive training in fully immersive virtual reality on visuospatial function and frontal-occipital functional connectivity in predementia: Randomized controlled trial. Journal of Medical Internet Research, 23(5), e24526. http://doi.org/10.2196/24526. PMCID: PMC8138710.

(Kwan et al., 2024) Kwan, R. Y. C., Liu, J., Sin, O. S. K., Fong, K. N. K., Qin, J., Wong, J. C. Y., & Lai, C. (2024). Effects of virtual reality motor-cognitive training for older peoplewith cognitive frailty: Multicentered randomized controlled trial. Journal of Medical Internet Research, 26(1), e57809. http://doi.org/10.2196/57809.

(Kwan et al., 2021) Kwan, R. Y. C., Liu, J. Y. W., Fong, K. N. K., Qin, J., Leung, P. K., Sin, O. S. K., Hon, P. Y., Suen, L. W., Tse, M., & Lai, C. K. (2021). Feasibility and effects of virtual reality motor-cognitive training in community-dwelling older people with cognitive frailty: Pilot randomized controlled trial. JMIR Serious Games, 9(3), e28400. http://doi.org/10.2196/28400.

(Li et al., 2025) Li, A., Qiang, W., Li, J., Geng, Y., Qiang, Y., & Zhao, J. (2025). Evaluating the clinical efficacy of an exergame-based training program for enhancing physical and cognitive functions in older adults with mild cognitive impairment and dementia residing in rural long-term care facilities: Randomized controlled trial. Journal of Medical Internet Research, 27, e69109. http://doi.org/10.2196/69109.

(Liao et al., 2020) Liao, Y.-Y., Tseng, H.-Y., Lin, Y.-J., Wang, C.-J., & Hsu, W.-C. (2020). Using virtual reality-based training to improve cognitive function, instrumental activities of daily living and neural efficiency in older adults with mild cognitive impairment. European Journal of Physical and Rehabilitation Medicine, 56(1), 47–57. http://doi.org/10.23736/S1973-9087.19.05899-4.

(Liao et al., 2019) Liao, Y.-Y., Hsuan Chen, I., Lin, Y. J., Chen, Y., & Hsu, W. C. (2019). Effects of virtual reality-based physical and cognitive training on executive function and dual-task gait performance in older adults with mild cognitive impairment: A randomized control trial. Frontiers in Aging Neuroscience, 11, 162. http://doi.org/10.3389/fnagi.2019.00162.

(Oliveira et al., 2021) Oliveira, J., Gamito, P., Souto, T., Conde, R., Ferreira, M., Corotnean, T., Fernandes, A., Silva, H., & Neto, T. (2021).

Virtual reality-based cognitive stimulation on people with mild to moderate dementia due to Alzheimer's disease: A pilot randomized controlled trial. International Journal of Environmental Research and Public Health, 18(10), 5290. http://doi.org/10.3390/ijerph18105290.
(J.-H. Park, 2022) Park, J.-H. (2022). Does the virtual shopping training improve executive function and instrumental activities of daily living of patients with mild cognitive impairment? Asian Journal of Psychiatry, 69, 102977. http://doi.org/10.1016/j.ajp.2021.102977.
(J.-H. Park et al., 2020) Park, J.-H., Liao, Y., Kim, D.-R., Song, S., Lim, J. H., Park, H., Lee, Y., & Park, K. W. (2020). Feasibility and tolerability of a culture-based virtual reality (VR) training program in patients with mild cognitive impairment: A randomized controlled pilot study. International Journal of Environmental Research and Public Health, 17(9), 3030. http://doi.org/10.3390/ijerph17093030.
(J. S. Park, 2020) Park, J. S., Jung, Y. J., & Lee, G. (2020). Virtual Reality-Based Cognitive-Motor Rehabilitation in Older Adults with Mild Cognitive Impairment: a Randomized Controlled Study on Motivation and Cognitive Function. Healthcare, 8, 335. http://doi.org/10.3390/healthcare8030335.
(Serino et al., 2017) Serino, S., Pedroli, E., Tuena, C., De Leo, G., Stramba-Badiale, M., Goulene, K., Mariotti, N. G., & Riva, G. (2017). A novel virtual reality-based training protocol for the enhancement of the "mental frame syncing" in individuals with Alzheimer's disease: A development-of-concept trial. Frontiers in Aging Neuroscience, 9, 240. http://doi.org/10.3389/fnagi.2017.00240. PMCID: PMC5529401.
(Thapa et al., 2020) Thapa, N., Park, H. J., Yang, J. G., Son, H., Jang, M., Lee, J., Kang, S. W., Park, K. W., & Park, H. (2020). The effect of a virtual reality-based intervention program on cognition in older adults with mild cognitive impairment: A randomized control trial. Journal of Clinical Medicine, 9(5), 1283. http://doi.org/10.3390/jcm9051283.
(H.-L. Yang et al., 2019) Yang, H.-L., Chu, H., Kao, C.-C., Chiu, H.-L., Tseng, I.-J., Tseng, P., & Chou, K.-R. (2019). Development and effectiveness of virtual interactive working memory training for older people with mild cognitive impairment: A single-blind randomised controlled trial. Age and Ageing, 48(4), 519–525. http://doi.org/10.1093/ageing/afz029.
(J. G. Yang et al., 2022) Yang, J. G., Thapa, N., Park, H. J., Bae, S., Park, K. W., Park, J. H., & Park, H. (2022). Virtual reality and exercise training enhance brain, cognitive, and physical health in older adults with mild cognitive impairment. International Journal of Environmental Research and Public Health, 19(20), 13300. http://doi.org/10.3390/ijerph192013300.

Table S3. Meta-analysis Results of Virtual Reality Interventions on Cognitive and Functional Outcomes

Outcome	Comparison	Studies (n)	Sample Size	SMD (95% CI)	P-value	I^2 (%)	P-heterogeneity
MMSE	A vs D	5	271	0.51 [0.06, 0.96]	0.03	69	0.01
	B vs C	3	349	0.62 [0.40, 0.83]	P< 0.00001	30	0.24
	B vs D	1	17	0.24 [-0.73, 1.21]	0.63		
MOCA	B vs C	5	444	0.40 [0.21, 0.59]	P< 0.0001	0	0.46
	A vs C	3	77	0.30 [-0.15, 0.75]	0.2	0	0.67
	A vs D	1	293	0.24 [0.01, 0.47]	0.04	-	-
	B vs D	1	20	0.24 [-0.64, 1.12]	0.6	-	-
CASI	B vs C	1	232	0.54 [0.28, 0.80]	P< 0.0001	-	-
	A vs D	1	60	1.65 [1.06, 2.24]	P< 0.00001	-	-
TMT-A	B vs C	2	86	-1.40[-1.88,0.93]	P<0.00001	0	0.84
	A vs D	2	134	-0.32[-0.66,0.03]	0.07	0	0.73
	A vs C	1	34	-0.09[-0.76,0.59]	0.8	-	-
TMT-B	B vs C	2	86	-1.29[-2.62,0.03]	0.06	87	0.006
	A vs D	2	124	-0.48[-0.84,0.12]	0.009	0	0.56
	A vs C	1	34	-0.47[-1.16,0.21]	0.18	-	-
SDST	A vs D	2	134	1.30 [0.92, 1.68]	0.01	86	0.007
DST-	B vs C	2	55	0.87 [-0.03, 1.78]	0.06	60	0.12
forward	A vs D	3	370	0.13 [-0.07, 0.34]	0.2	0	0.47
DST-	B vs C	2	101	0.56 [0.16, 0.96]	0.006	0	0.35
backward	A vs D	2	314	-0.04[-0.26,0.18]	0.71	0	0.96
SCWT	A vs C	1	34	0.13 [-0.55, 0.80]	0.71	-	-
	A vs D	1	293	0.08 [-0.15, 0.31]	0.51	-	-
VFT	B vs C	2	71	0.23 [-0.95, 1.42]	0.7	80	0.02
	A vs D	1	21	0.00 [-0.86, 0.86]	1	-	-
FAB	B vs C	1	20	0.43 [-0.46, 1.32]	0.34	-	-
	B vs D	1	17	0.19 [-0.78, 1.16]	0.7	-	-

IADL	A vs C	2	60	0.38 [-0.13, 0.90]	0.14	0	0.58
	B vs D	2	49	0.50 [-0.08, 1.08]	0.09	46	0.17
FFP	A vs C	1	17	0.00 [-0.95, 0.95]	1	-	-
	A vs D	1	293	-0.31[-0.54,0.08]	0.008	-	-
TUG	B vs C	1	60	-0.59[-1.11,0.08]	0.02	-	-
	A vs C	1	17	0.30 [-0.66, 1.26]	0.54	-	-
	B vs D	1	20	0.05 [-0.82, 0.93]	0.91	-	-
	A vs D	1	293	-0.18[-0.41,0.05]	0.13	-	-
Grip	A vs D	1	68	0.29 [-0.19, 0.77]	0.23	-	-
Strength	B vs C	1	60	0.53 [0.02, 1.05]	0.04	-	-
SGDS	A vs C	1	26	0.53 [-0.26, 1.32]	0.19	-	-
	A vs D	2	62	-0.16[-0.66,0.34]	0.53	0	0.87

Group Definitions: A: Fully immersive VR；B: Partially immersive VR；C: Active control；D: Passive control；Statistical Conventions: SMD: Standardized Mean Difference; positive values favor the VR group；Bold text: Statistically significant results ($P < 0.05$) I^2: Heterogeneity index; values ≥50% indicate substantial heterogeneity；–: Not applicable (single-study comparisons).
